# Mindfulness, cognition, and long-term meditators: Toward a science of advanced meditation

**DOI:** 10.1162/IMAG.a.82

**Published:** 2025-07-18

**Authors:** Sebastian Ehmann, Idil Sezer, Isaac N. Treves, John D.E. Gabrieli, Matthew D. Sacchet

**Affiliations:** Meditation Research Program, Department of Psychiatry, Massachusetts General Hospital, Harvard Medical School, Boston, MA, United States; Department of Psychology, University of Arizona, Tucson, AZ, United States; Center for Consciousness Studies, University of Arizona, Tucson, AZ, United States; Paris Brain Institute, Sorbonne University/CNRS/INSERM, Paris, France; Department of Brain and Cognitive Sciences, Massachusetts Institute of Technology, Cambridge, MA, United States; McGovern Institute for Brain Research, Massachusetts Institute of Technology, Cambridge, MA, United States

**Keywords:** mindfulness meditation, long-term meditators, advanced meditation, cognition, emotion regulation, awareness

## Abstract

Mindfulness meditation is a systematic training in equanimity, sensory clarity, and concentration rooted in ancient contemplative traditions. Here, we synthesized cognitive–behavioral outcomes in long-term meditators (LTMs) resulting from diverse, prolonged meditation practices. Preliminary evidence suggests that LTMs exhibit increased cognitive–sensory integration and decoupling of affective processes, demonstrated by enhanced interoceptive awareness, reduced negative affective pain perception, and more rational decision making. Additionally, LTMs may experience more emotional neutrality, malleable self-boundaries, and altered self-awareness. Neuroimaging findings included increased bottom-up activation, particularly within the salience network (interoception, pain, affect), and reduced connectivity between the executive (dorsolateral prefrontal cortex) and salience (dorsal anterior cingulate cortex) networks (reduced pain). The literature also suggests reduced fear and amygdala activation (mitigated negative affect), increased temporoparietal junction activation (pre-reflective experiential processes, empathy), and altered midline default-mode network activation, which was associated with emotional neutrality and non-ordinary states of consciousness. Methodological limitations restricted the interpretation of trait effects, emphasizing the need for a unified framework to systematically investigate advanced meditation’s states, stages, and endpoints using neurophenomenology. In summary, LTMs display a distinct neurophenomenological gestalt of mindfulness, wherein meditative expertise is reflected in enhanced cognitive flexibility and integration, self-regulation, and non-dual awareness—signifying a potentially important form of embodied cognition.

## 1 Introduction

### 1.1 Meditation and mindfulness

Mindfulness is a fundamental mental capacity that has been practiced for millennia in various contemplative branches of Hinduism, Buddhism, and other traditions. Its roots trace back to the *Satipatthana*, a core Buddhist meditation system described in the *Pāli Canon*, the oldest surviving Buddhist scriptures (Bodhi, 2011). To practice according to the *Satipatthana*, two factors are emphasized, *sati* and *sampajañña*, translating from Pali, the liturgical language of Theravada Buddhism, into “mindfulness” and “clear comprehension” (Sharf, 2014). In Buddhism, mindfulness is understood as “lucid awareness,” which, when cultivated with clear comprehension, contributes to the development of insight into the “nature of how things are” (Bodhi, 2011). The intention behind diligently practicing mindfulness is to cultivate the core mental faculties involved in insight (Sugunasiri, 2008), gradually reducing unwholesome mental actions and, in turn, suffering (Ingram, 2018).

These soteriological origins of mindfulness meditation paved the way for modern scientific definitions that extracted mindfulness from its traditional context. Kabat-Zinn (1994) popularized mindfulness as the intentional and nonjudgmental act of paying attention in the present moment, which served as the basis of Mindfulness-Based Stress Reduction (MBSR; Kabat-Zinn, 1994, 2013) and other evidence-based mindfulness-based programs (Garland, 2013; Segal et al., 2013; Witkiewitz et al., 2013) and therapies (Ost, 2014; Panos et al., 2014). Collectively, there is strong evidence that mindfulness practices can be used to treat substance abuse (Goldberg et al., 2018; Priddy et al., 2018), depression (Goldberg et al., 2018; Hofmann et al., 2010; Hoge et al., 2023; Khoury et al., 2013; Piet & Hougaard, 2011; Strauss et al., 2023), anxiety (Hofmann et al., 2010; Khoury et al., 2013), stress (Khoury et al., 2013), pain (Goldberg et al., 2018), post-traumatic stress (PTSD; Boyd et al., 2018), attention-deficit/hyperactivation disorder (ADHD; Poissant et al., 2019), eating disorder (Wanden-Berghe et al., 2011), and smoking (Goldberg et al., 2018).

Outside clinical contexts, mindfulness is increasingly employed for emotional regulation and enhanced well-being (Pepping et al., 2016). This broad transdiagnostic efficacy has driven renewed interest in understanding the underlying cognitive and neural mechanisms, particularly among advanced practitioners (Sacchet et al., 2024).

#### 1.1.1 A broader framework of mindfulness for meditation practices

To reduce conceptual ambiguities across meditative traditions and between traditional and secular definitions, Young (2016) developed a multidimensional framework of mindfulness that aims to capture specific mental skills that are trained across meditative practices. This model distills mindfulness into three interpenetrating core skills: Concentration, Sensory Clarity, and Equanimity. Concentration refers to the ability to focus attention on a chosen object; Sensory Clarity is the capacity to discern the fine details of sensory experience; and Equanimity involves maintaining non-reactivity toward experiences as they arise and pass away. These three dimensions are not only distinct but also mutually reinforcing, providing a structured way to understand how mindfulness training cultivates attentional control and perceptual sensitivity across different practices.

This review adopts this framework as its theoretical model for its inclusivity across diverse contemplative traditions. It enables systematic analysis of how different meditative practices cultivate the core domains of Concentration, Sensory Clarity, and Equanimity, addressing conceptual fragmentation with a structured, skill-based approach to understanding meditation’s cognitive and neural mechanisms. Skill-based models are also more suitable for understanding meditative development, which we will address in subsequent sections.

This review synthesizes behavioral changes and neurocognitive mechanisms, drawing on evidence from long-term meditators (LTMs). We begin by outlining the theoretical relationship between cognition and mindfulness meditation, followed by an exploration of experienced meditators as key to understanding mindfulness’s long-term effects. We introduce a conceptual distinction between advanced meditators (AMs) and LTMs to contextualize our findings within a third wave of meditative science focused on deep cognitive development and its neural correlates. Finally, we present two additional integrative frameworks to guide our analysis.

### 1.2 Cognition and mindfulness

Cognition is a multifaceted construct comprising various domains, including sensation, perception, motor skills, attention, memory, executive functioning, processing speed, and language (Harvey, 2019). Its evolutionary development has been tied to the expansion and structural organization of “association cortices,” which have been subject to strong selective pressures during recent hominid evolution (Yeo et al., 2011). These cortices form the bulk of the cerebral cortex, with the recent expansion of the parietal region proposed as central to the distinctive cognitive abilities of humans (Bruner & Lozano, 2015; Bruner et al., 2014).

Psychophysiological models of cognition have shifted from localized signal computations (*Sherringtonian view*) to distributed processes within representational spaces by neural populations (*Hopfieldian view*), where specific neuron details are secondary to network dynamics (Barack & Krakauer, 2021; Hopfield, 1982, 1984; Hopfield & Tank, 1986; Sherrington, 1906). According to Bayne et al. (2019), cognition is structured around three key principles: the recombination of representations for concept and model generation, adaptive information management spanning logical reasoning and perceptual inferences, and sensory processing to guide behavior. These processes operate hierarchically, with abstract cognitive functions unfolding over longer timescales (Laukkonen et al., 2025; Northoff & Zilio, 2022).

Recent empirical work supports this hierarchical organization. The brain appears to operate across intrinsic neural timescales, where higher-order regions—such as the default mode network (DMN)—maintain longer temporal integration windows than unimodal sensory areas (Friston, 2008; Taylor et al., 2015; Wolff et al., 2022). This temporal hierarchy aligns with spatial gradients in brain organization, placing primary sensory areas and transmodal regions such as the DMN at opposite ends of an abstraction spectrum (Margulies et al., 2016). Notably, this structure remains stable across both resting and task states, underscoring its robustness in cognitive processing (Golesorkhi et al., 2021).

The Free Energy Principle (FEP) reflects these characteristics, positing that cognition operates through predictive processing, where the brain continually refines its expectations by minimizing prediction errors between anticipated and actual sensory input (Friston, 2010; Bubic et al., 2010). This involves reconciling top-down predictions with incoming bottom-up sensory data. Cognitive agents can either update internal models constituted of higher-level priors, such as the self (Friston, 2018), or act upon these inferences by extending cognition onto the external environment (“active inference”; Constant et al., 2022). In meditation, mitigated physical activity and voluntary attention modulation increase present-moment awareness by reducing the weighting of higher-order predictions in favor of bottom-up input (Lutz et al., 2019). This shift is hypothesized to gradually minimize prolonged temporal processing, enabling the deconstruction of habitual self-referential patterns (Dahl et al., 2015; Friston, 2018; Laukkonen & Slagter, 2021).

In contrast, *Enactivism*—influenced by contemplative traditions—views cognition as an embodied, relational process emerging from direct interaction with the environment, rather than representational modeling (Thompson, 2007; Varela et al., 1992). Unlike FEP, enaction emphasizes *sense-making* and *agency* as fundamentally rooted in lived experience, with critics arguing that FEP misrepresents these concepts (Di Paolo et al., 2022). Enactive approaches to meditation describe a progression from adaptive sense-making to non-dual reflexive knowing, where cognition shifts from being entangled in habitual sense-making to becoming aware of its own processes, enabling a more fluid, direct engagement with experience (Meling, 2021, 2022).

The growing interest in mindfulness has shifted the scientific focus from its efficacy to exploring its mental and neurocognitive mechanisms. Early research identified four key components: intention, attention, and attitude, collectively affording re-perception (Shapiro et al., 2006). More recent work emphasizes self-processing, in which mindfulness meditation enhances self-awareness, self-regulation, and self-transcendence by improving neurocognitive processes (Vago & Silbersweig, 2012). These improvements include enhanced meta-cognition and attention regulation, body awareness, emotion regulation, and shifts in self-perspectives (Coffey et al., 2010; Dorjee, 2016; Hölzel et al., 2011; Tang et al., 2015; Vago & Silbersweig, 2012), with synthesizing research suggesting shifts toward a more embodied and flexible self as the two key meta-mechanisms (Christoff et al., 2011; Giommi et al., 2023; Moore & Malinowski, 2009). Notably, the mindfulness mechanisms behind these changes appear similar for both non-meditators and meditators (Burzler et al., 2019).

### 1.3 The study of long-term and advanced meditators

LTMs represent a unique population for studying meditation-induced alterations in cognitive function due to their extensive training. Their deep development within specific meditation practices facilitates strong neurocognitive effects, offering precise insights into how sustained mental training shapes brain processes (Slagter et al., 2011). However, while LTMs provide valuable insights into neurocognitive changes associated with meditation, it is crucial to recognize potential limitations in generalizability due to inherent differences from the general population.

Sustainable, long-term transformations are hypothesized to unfold through a natural progression from transient cognitive states to persistent cognitive traits, largely dependent on the practitioner’s expertise (Goleman & Davidson, 2017). States and traits differ in both temporal scale and locus of origination: states are brief and influenced mainly by external circumstances, while traits are persistent, durable, and internally caused (Chaplin et al., 1988). During mindfulness meditation, practitioners experience mindful states, which, with continuous effort, likely stabilize into trait-level changes (Atasoy et al., 2023; Bauer et al., 2019; Kiken et al., 2015). This so-called trait mindfulness is typically measured through self-report questionnaires and reflects a person’s dispositional mindful aptitude (Baer et al., 2004, 2006, 2008; MacKillop & Anderson, 2007; Walach et al., 2006). Increases in state mindfulness during interventions predict corresponding shifts in trait mindfulness (Kiken et al., 2015), and trait scores correlate with meditation experience (Vinchurkar et al., 2014). However, trait mindfulness shows inconsistent links with attentional performance (Quickel et al., 2014), and certain measures fail to distinguish between experienced monks and novices (Christopher et al., 2009).

While mindfulness-based programs and continuous practice may yield positive effects on well-being, these changes are often limited in both duration and magnitude. More profound psychological transformations are thought to require extensive, consistent practice, which traditional meditative frameworks describe as developmental stages leading to transformative shifts (Berkovich-Ohana et al., 2013, 2017; Dor-Ziderman et al., 2016; Ehmann et al., 2025; Goleman & Davidson, 2017; Hagerty et al., 2013). This may point to a notable conceptual distinction between LTMs and what we refer to as AMs (Sacchet et al., 2024).

#### 1.3.1 Operationalizing advanced meditation

Although LTMs provide valuable insights into meditation-induced cognitive changes, we propose that AMs constitute a distinct but overlapping category. Unlike LTMs, whose expertise is typically assessed through duration-based metrics (e.g., total meditation hours), AMs are distinguished by their experience, including qualitative shifts in skill that enable practice of deeper states and traits. These include absorptive concentrative meditation states (e.g., what have been called *jhana*; Chowdhury et al., 2024; Demir et al., 2025; Ganesan et al., 2024; Potash et al., 2024, 2025; Sparby & Sacchet, 2024; Treves et al., 2024b; Yang et al., 2023, 2024a), advanced investigative insight meditation (AIIM) states (Yang et al., 2024b), and meditative endpoints including non-dual awareness and cessations of consciousness (Agarwal & Laukkonen, 2024; Chowdhury et al., 2023; Laukkonen et al., 2023; van Lutterveld et al., 2024). Such shifts may be marked by deep equanimity, refined sensory clarity, improved attention and self-regulation, and greater cognitive flexibility both during and outside of meditation (Berkovich-Ohana et al., 2024; Young, 2016). Emerging evidence suggests that skill-based proficiency toward experiencing these states correlates more strongly with well-being than simple duration-based expertise (Nair et al., 2018).

Consequently, we conceptualize advanced meditation as the progressive unfolding of states and stages that develop with mastery and sustained practice (Sacchet et al., 2024). Meditative development within this framework includes nonlinear trajectories, potentially culminating in meditative endpoints—landmark experiences and potential shifts in consciousness and self-perception (Berkovich-Ohana et al., 2024; Chowdhury et al., 2023; Galante et al., 2023; Sacchet et al., 2024; van Lutterveld et al., 2024). Our recent work has translated traditional meditative concepts of states and stages into scientific language, enabling a rigorous interdisciplinary study of meditative development (Galante et al., 2023; Wright et al., 2024), in which we uncovered new connections between changes in consciousness observed in advanced meditative states and related neural and physiological mechanisms (Chowdhury et al., 2023, 2024; Demir et al., 2025; Ganesan et al., 2024; Potash et al., 2024, 2025; Sezer & Sacchet, 2025; Treves et al., 2024b; van Lutterveld et al., 2024; Wright et al., 2024; Yang et al., 2023, 2024a, 2024b).

Studying AMs may thus provide critical insights into meditative development, offering novel perspectives for both clinical and non-clinical applications in mental health and well-being. However, further work is needed to sharpen the operational distinction between LTMs and AMs, focusing on clear criteria for mastery, including phenomenological access to traditional meditative states, stages, and endpoints, alongside the development of novel objective assessments, such as behavioral stress tests for equanimity and enhanced interoceptive sensitivity measures.

#### 1.3.2 Classifying meditation practices and states

A central challenge in the scientific study of mindfulness meditation, particularly in advanced states and stages, lies in effectively categorizing distinct meditative techniques. Earlier research proposed a “phenomenological matrix” that identifies three core dimensions: object orientation, de-reification, and meta-awareness (Lutz et al., 2015). Building on this, we introduced an *activity-based phenomenological classification system* that integrates meditative practices with their corresponding phenomenological experiences. This structured approach advances our understanding of meditation by categorizing practices based on both the *type of activity* (e.g., active vs. receptive) and the *content of awareness* (e.g., object based vs. awareness based) (Sparby & Sacchet, 2022). Our framework identifies four core meditative activities: *focusing* and *releasing*, as well as *imagining* and *moving*. Each pair reflects a distinct activity mode, either *receptive observation* or *active production*. These activities are further unified under a broader umbrella of passive or active *awareness* of a meditative object, categorized into six sensory domains: (1) *thought*, (2) *sound*, (3) *sight*, (4) *taste*, (5) *smell*, and (6) *bodily sensations*. These represent the *content of awareness*, while the specific meditative activity describes the *method of engagement* with that content.

For example, in Theravadan Buddhist *samatha* meditation, practitioners concentrate on a specific object, such as the breath at the nostrils (Tiwari, 1988). With increasing expertise, meditators may transition to states devoid of object-based awareness, described as pure consciousness events. These are characterized by the dissolution of typical subject–object distinctions (Woods et al., 2020, 2022) and are often associated with experiences of self-transcendence or unity (Aron et al., 1992). Concentration-based absorptive states like these are frequently referred to as *samādhi*—a term signifying “a state of being firmly fixed,” marked by profound mental singularity and stability (Sraman, 2002). Importantly, *samādhi* is only one of many potential non-ordinary states of consciousness that can be achieved through meditative practice.

During pure consciousness events, a subtle form of subject–object dichotomy still persists, as consciousness itself becomes the meditative object. This state represents a refined yet partial dissolution of dualistic perception. The final step in unifying practice activities and meditative objects is described as *awareness-of-awareness*, where awareness is non-propositionally, reflexively aware of itself without mediation, fully collapsing the subject–object distinction into non-duality (Dunne et al., 2019; Josipovic, 2021; Lutz et al., 2007; Meling, 2021, 2022; Sparby & Sacchet, 2022).

This progressive refinement from object-based meditative methods to nondual awareness reflects an expertise-dependent unfolding. Here, awareness transitions from implicit (dualistic, object-focused) to explicit (nondual, reflexive awareness), mirroring the stages of the activity-based phenomenological classification system (Josipovic, 2019, 2021, 2024; Josipovic & Miskovic, 2020; Sparby & Sacchet, 2022). This developmental trajectory not only captures the phenomenological shifts observed in advanced meditative states but also aligns with empirical descriptions of meditative expertise (Cooper et al., 2022). Our framework is the first to systematically classify these diverse meditation techniques based on empirical descriptions (Matko et al., 2021) and contemplative traditions (Anālayo, 2020a), representing a significant step toward resolving semantic ambiguities surrounding the definition and empirical understanding of mindfulness and its diverse outcomes.

The following section addresses the need for a unified framework to support systematic scientific exploration of LTMs and AMs.

### 1.4 An empirical framework for mindfulness research

Since 2006, contemplative science has grown exponentially, with over 16,000 scientific articles published since 1966 (Baminiwatta & Solangaarachchi, 2021). This surge underscores the need for a unified empirical framework to systematically investigate mindfulness. Key challenges include semantic inconsistencies that create construct ambiguity, divergent self-report measures with unclear definitions and interpretations (Bergomi et al., 2013; Grossman & Van Dam, 2011), methodological limitations in research design (Van Dam et al., 2018), and insufficient exploration of advanced meditative states and stages (Galante et al., 2023). The limited exploration of advanced meditation has resulted in overlooking unique benefits and potential challenges, which have been called adverse events, associated with meditative development, which might include emotional distress, cognitive difficulties, physical discomfort, and, in intensive retreat settings, even severe reactions such as mania or psychosis (Cebolla et al., 2017; Galante et al., 2023; Goldberg et al., 2020; Lindahl et al., 2017; Sacchet et al., 2024; Schlosser et al., 2019). There is still an ongoing debate on the nature of challenging experiences in meditation, including whether they are all negative or also provide long-term benefits, such as growth and resilience (Sparby & Sacchet, 2025).

To address these limitations, we recently established an empirical framework for investigating mindfulness and development into what we have termed meditative development (Galante et al., 2023). By integrating Young’s (2016) mindfulness model and the *Activity-Based Phenomenological Classification System* (Sparby & Sacchet, 2022) into this framework, we enable a multi-layered analysis that captures the distinct mental skills cultivated through meditation practice, their phenomenological expressions, and their developmental trajectories over time.

Galante et al.’s (2023) overarching framework emphasized the need for longitudinal, interdisciplinary, transparent, and ontologically agnostic research. It advocates for integrating contemplative scholastic work, such as traditional stages of insight (Grabovac, 2015), with context-sensitive, multi-method research approaches. This aims to address a growing body of research indicating that mindfulness can have adverse or challenging effects (Aizik-Reebs et al., 2021; Lambert et al., 2021), especially when practiced alone, in secular contexts, and without developmental frameworks (Cebolla et al., 2017; Schlosser et al., 2019). Additionally, direct collaboration with meditation practitioners could enhance empirical rigor by enabling a deeper understanding and systematic assessment of key factors, such as practice history, specific meditation methods, experiential patterns, and the socio-cultural contexts of practice (Matko et al., 2021; Sparby & Sacchet, 2022).

Together, these three frameworks (Galante et al., 2023; Sparby & Sacchet, 2022; Young, 2016) offer a coherent empirical foundation for studying mindfulness across varying levels of expertise and practice types. This integrated approach enables a clearer understanding of cognitive processing by linking perceptual adaptations to specific behavioral patterns and brain changes, advancing neurophenomenological research in contemplative science.

## 2 Methods

This review presents a comprehensive synthesis of the relationships between LTMs, behavior, and cognition, alongside a selective overview of related neuroscience research. Relevant studies of LTMs were identified based on a minimum threshold of 1,500 hours of meditation experience for inclusion. While there is no universally agreed-upon definition for LTMs, many studies generally consider several years or at least 1,000 hours of meditation as sufficient for inclusion (Wipplinger et al., 2023). Our criterion of 1,500 hours reflects approximately 1 hour of daily practice over 5 years, providing a practical and literature-supported benchmark for significant neurocognitive and behavioral changes. Study selection did not specifically filter for AMs due to the paucity of clear, phenomenological, skill-based descriptions of experienced meditators in most studies; however, these issues were addressed through discussion in the relevant sections.

**Fig. 1.**
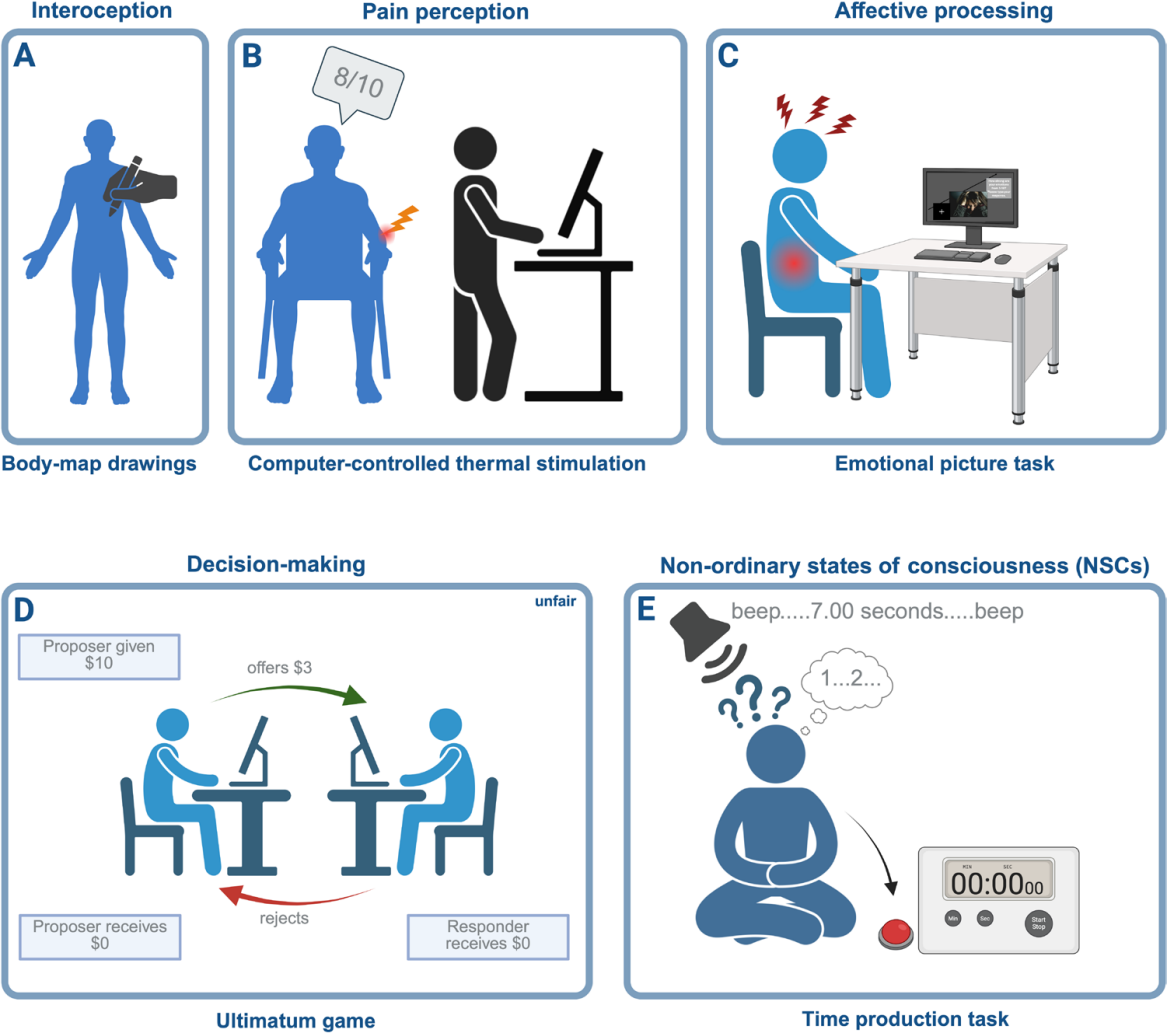
Sample cognitive–behavioral tasks employed within the different domains. *Note*. Five exemplary tasks used in the study: (A) Cardiac interoceptive awareness was assessed by having participants mark where they felt their heartbeat on a body map, generating proportional maps based on these responses (Khalsa et al., 2020). (B) Graded thermal stimulation induced moderate pain, which participants rated on a point scale (Lutz et al., 2013; Grant & Rainville, 2009; Grant et al., 2010, 2011; Perlman et al., 2010; Zorn et al., 2020). (C) Participants viewed emotional pictures and rated their subsequent emotional responses (Chen et al., 2018; Taylor et al., 2011). (D) The Ultimatum Game assessed economic decision-making, with participants deciding whether to accept or reject various offers from human and computer partners (Kirk et al., 2011). (E) In the time production task, participants pressed a button to match target durations, evaluating their time estimation ability with eyes closed (Berkovich-Ohana et al., 2011).

The review is separated into four main chapters encompassing different but interpenetrating cognitive domains: perception, emotional processing, higher-order functions, and non-ordinary states of consciousness. Sample tasks from each section are represented in Figure 1. In the discussion, we will address methodological limitations, summarize and contextualize the observed behavioral trait effects, and synthesize findings within an overarching dynamical self-processing model.

This article is a review of previously published studies and does not involve any new data collection with human participants or animals. As such, no institutional ethics approval was required. The authors have adhered to ethical guidelines for academic writing and research integrity by accurately citing all sources and ensuring that the content presented respects the intellectual property of the original authors. Any potential biases in the selection and interpretation of studies have been minimized, and the authors declare no conflicts of interest related to this manuscript.

## 3 Cognitive Functions in Long-Term Meditators

### 3.1 Somatosensory perception

Attention involves the process of focusing on relevant information, and perception is the cognitive process by which sensory information is interpreted and transformed into a representation that captures the context of the scene (Albright, 2015). This transformative process of perception involves neuronal computation that assigns semantic interpretations to incoming signals, leveraging contextual information to enhance the understanding and meaning of sensory input. Consequently, perception can be consistently broadened and enriched through systematic conceptual development (Newen, 2017). This aligns with a predictive processing account, in which moment-to-moment conscious perception is shaped through the constant updating of higher-level priors or through action, while also accommodating higher-level features like emotions and person impressions (Binder et al., 2017; Kahl & Kopp, 2018). There is some resonance between neuroscientific definitions and Buddhist understanding of perception. In Pali, Saññā (perception) denotes the discernment of objects from sense signals (Wright et al., 2023). It constitutes an integral element of the Buddhist doctrine of “The Five Aggregates of Clinging,” which elucidates how people turn neutral sense information into experiences of suffering and distress. Consequently, changes in ordinary perception are at the core of Buddhist soteriological frameworks and are viewed as a pivotal mechanism within mindfulness practices (Shapiro et al., 2006). Substantial perceptual alterations, including those experienced in advanced meditation, might predominantly manifest in adept meditators or under intense practice regimens, an aspect that has garnered limited scientific exploration. Qualitative investigations involving accomplished Burmese meditators revealed four modifications in perception: (1) heightened perceptual sensitivity, (2) diminished subject–object dichotomous perception, (3) recognition of interconnected perceptual processes, and (4) nonconceptual perception (Full et al., 2013). These shifts toward non-conceptual cognition are suggested to facilitate insights into a deeper reality (Bodhi, 2011). However, it is important to distinguish this from accessing an “ultimate reality” (Kant, 1787/1998) or overcoming human perceptual illusions (Carbon, 2014). Instead, it represents a progression toward a less biased mental state, characterized by the disentanglement from habitual cognitive discriminations (i.e., biased attentional sampling), thus allowing more resources for direct sensory discrimination (Thompson, 2023). Indeed, intensive meditation training has been linked to enhanced perceptual discrimination, afforded by increased vigilance and perceptual sensitivity, leading to reduced cognitive load and increased sustained attention (MacLean et al., 2010). Numerous studies have examined perceptual changes in LTMs in both clinical and non-clinical contexts. We will next describe two studies on interoceptive awareness and six studies on pain perception.

#### 3.1.1 Interoceptive awareness

Recent studies proposed a neurocognitive hierarchy of self-processing, in which body–environment information follows a gradual integration from interoceptive processing to mental self-processing (Qin et al., 2020). For example, research has demonstrated that interoceptive signals can modulate the perception of the external world (exteroception), as experienced through body surfaces (somatosensory perception; Al et al., 2021). Interoceptive awareness is the conscious perception of bodily signals that collectively create a physiological sense of self, encompassing sensations such as breathing, heartbeat, hunger cues, and the physical manifestations linked to emotions (Barrett et al., 2004; Cameron, 2001; Craig, 2002; Vaitl, 1996). A critical neural substrate for interoception is the insula (Qin et al., 2020), with other implicated regions including the anterior cingulate cortex (ACC), posterior cingulate cortex (PCC), medial prefrontal cortex (mPFC; Treves et al., 2024a), and temporoparietal junction (TPJ; Hölzel et al., 2011).

The cultivation of interoceptive awareness may be a foundational mechanism in *vipassanā* or mindfulness meditation, with body sensations often used as the objects of meditation in various practice traditions. For instance, a practice in the *vipassanā* tradition guides meditators to methodically scan their bodies, attentively observing bodily sensations without judgment or the intent to modify them. Within the *kāyagatāsati* sutta, mindfulness is cultivated by practicing body awareness with several exercises, such as noting postural or movement differences, or meditating upon the bodies’ constituents (Anālayo, 2020a, 2020b). In the yogic traditions, *asanas* guide the practitioners’ bodies and awareness into specific postures to create relaxation for further meditative practices (Iyengar, 1995). Thus far, mindfulness meditators have exhibited enhanced insular connectivity, increased cortical thickness in the right anterior insula and TPJ gray matter, and either enhanced or reduced right anterior insular activity, potentially reflecting an improved ability to modulate body awareness according to the valence of stimuli (Farb et al., 2007, 2013, 2015; Gibson, 2019; Haase et al., 2016; Hölzel et al., 2011; Todd & Aspell, 2022). Research indicates that greater insular cortical thickness is consistently associated with higher mindfulness scores, with some evidence also linking it to enhanced body awareness (Friedel et al., 2015; Treves et al., 2019, 2024a). Notably, the “observe” facet of the FFMQ self-report questionnaire may be particularly sensitive to interoceptive changes in mindful individuals (Hölzel et al., 2011). In this context, mindfulness meditation has been suggested as a potential intervention to assist with affective and psychosomatic disorders that encompass interoceptive distortions within their underlying pathology (Farb et al., 2015).

To test the effects of meditation on interoception, Fox et al. (2012) enrolled 38 meditators with an average of 11 years and 2,051 hours of meditation experience, spanning from 1 to 15,000 hours. The researchers divided the sample into four quartiles, with the bottom and top quartiles representing novice (average meditation experience = 28 hours, *SD* = 24 hours) and LTMs (average meditation experience = 7,231 hours, *SD* = 4,410 hours), respectively. Interoceptive accuracy was assessed by correlating meditators’ subjective ratings of bodily sensations during a body-scan meditation with objective measures of tactile sensitivity. Participants rated sensations across 20 body regions, which were compared with a composite Somatic Sensitivity Rank (SSR). The SSR combined psychophysical two-point discrimination thresholds and cortical representation in the primary somatosensory cortex, derived from the literature and validated through neurosurgical and neuroimaging data. The correlation between subjective ratings and SSR quantified participants’ ability to accurately perceive and report bodily sensations. Participants were familiar with the body-scan meditation type, but their experience varied widely and represented only a fraction of their overall meditation practice (less than 10%).

Results included a large difference in trait introspective accuracy between LTMs and beginner meditators. Furthermore, total body-scan experience, meditation experience, and average practice amount per month predicted introspective accuracy. A dose–response relationship was observed between meditation experience and interoception. The significance of this relationship remained even after accounting for the participants’ body-scan experience in their overall meditation practice. Nevertheless, this study design precluded the exploration of the relationship between subjective and objective measures of somatosensory awareness, underscoring the need for future research to examine changes in subjective intero-exteroceptive awareness and brain topography in LTMs.

Conflicting evidence was reported by Khalsa et al. (2008) for cardiac interoceptive accuracy during rest and physiological arousal (Khalsa et al., 2020). In their first study, participants performed a heartbeat detection task that involved presenting auditory tones to participants. These tones were either simultaneous or non-simultaneous with the participants’ actual heartbeats. Participants had to judge whether each tone coincided with their perceived heartbeat sensation, without being allowed to take their pulse. To address previous methodological limitations, in the second study, participants received blinded infusions of isoproterenol—a beta-adrenergic drug similar to adrenaline—at various doses and saline while dial rating their internal body sensations in real time, followed by retrospective ratings of physical sensations and emotional experiences. Interoceptive detection accuracy was assessed by calculating cross-correlations between participants’ dial ratings and their actual heart rate changes during each infusion, with higher correlations indicating greater accuracy in perceiving bodily sensations. This approach aimed to enhance physiological arousal by stimulating the sympathetic nervous system and intensifying internal sensations, potentially counteracting the low detection rates observed in quiescent resting-state scenarios. Cardiac spatial interoceptive awareness was assessed using drawings on a two-dimensional paper manikin, where participants indicated areas of felt heartbeat sensations. These drawings were digitized and converted into proportional body maps for specific doses, with each pixel value representing the proportion of participants reporting sensation in that area. While not explicitly stated, accuracy was likely inferred from the consistency and anatomical relevance of reported sensations across participants. Other assessments included self-reported ratings and electrocardiogram (EKG).

The researchers recruited 15 LTMs and 15 matched non-meditators. The meditators averaged 10.8 years and 4,947 hours of meditation experience, with 19 days completed in a retreat setting. Eleven meditators practiced *vipassanā*, while four engaged in Kundalini meditation, which combines *active imagination*, breathing techniques, mantras, and *passive observe-and-release* activities, suggesting familiarity with interoceptive meditations for most meditators. No between-group differences were found for cardiac interoceptive awareness. These outcomes were further validated through a subsequent meta-analysis, which explored cardiac interoceptive awareness between meditators and non-meditators from various traditions and indicated that meditation may not substantially impact cardiac interoceptive awareness, consolidating the researchers’ conclusions.

In summary, preliminary research suggests that LTMs may exhibit improved interoceptive awareness compared with non-meditating controls across various practices, including body-scan meditation. Nevertheless, these effects are not observed in cardiac interoceptive awareness, underscoring the need for further investigation, including longitudinal studies that directly compare different meditation techniques.

#### 3.1.2 Pain perception

Pain is a distressing sensory and emotional phenomenon intricately intertwined with various cognitive domains, sociocultural influences, and biological determinants (Binder et al., 2011; Khera & Rangasamy, 2021; Ossipov et al., 2010; Symbaluk et al., 1997) and processed in the “pain matrix,” including the anterior cingulate cortex (ACC), insula, thalamus, primary (S1) and secondary somatosensory (S2) cortices, amygdala, periaqueductal gray, and prefrontal cortices (PFC; Ingvar, 1999; Rainville, 2002; Tracey & Mantyh, 2007). Recent resting-state functional connectivity (rsFC) research has identified a distinct pain network comprising the dorsolateral prefrontal cortex (dlPFC), anterior insula, thalamus, precuneus, and ACC (Lee et al., 2019), which strongly overlaps with the salience network (SN) due to the high salience of painful stimuli (Borsook et al., 2013). Pain perception is complex, as sensory pain, a characterizable and localizable physiological response to harmful stimuli, is reciprocally intertwined with a cognitive–affective dimension that reflects the emotional and motivational appraisal of the stimuli (Grant, 2014; Price, 2002). Sensory-discriminative aspects of pain involve the somatosensory cortices, thalamus, and posterior insula (Coghill et al., 1999), while affective-motivational components are processed in the dorsal ACC and anterior insula (Rainville et al., 1997), with cognitive components localized in the PFC (Strigo et al., 2002).

Compared with a neuroscientific perspective, Buddhism conceives of pain within the broader context of suffering, wherein sensory experiences are modulated by approach and avoidance tendencies through the grasping of more pleasant goal states (Thompson, 2023; Waikakul & Waikakul, 2016). Meditation aims to reduce grasping by transforming cognitive processes to re-perceive sensory input as less distressing (Waikakul & Waikakul, 2016). Functionally, meditation introduces a divide between the sensory experience and its cognitive interpretation through acceptance, non-judgment, and decentering, often encapsulated within the overarching construct of equanimity (Desbordes et al., 2014; Soler et al., 2021).

Neuroimaging research employing functional magnetic resonance imaging (fMRI) and positron emission tomography (PET) has demonstrated that mindfulness meditation-induced pain relief is associated with increased activation in sensory and affective core pain areas such as the ACC, S2, and insula, reduced activation in the thalamus, amygdala, PCC, periaqueductal gray, and hippocampus (Tang et al., 2015; Wipplinger et al., 2023), as well as enhanced cortico-thalamic regulation (Riegner et al., 2023) and increased within-SN connectivity between the anterior insula and dorsal ACC (Sezer et al., 2022). Crucially, preliminary evidence suggests that LTMs reduce pain unpleasantness more effectively than novices by deactivating appraisal-related regions like the PFC, while novices rely on cognitive control strategies due to their inability to selectively modulate affective and sensory domains (Grant, 2014; Wipplinger et al., 2023).

Neuro-mechanistic studies further support these findings, pointing to a mindfulness-induced dissociation of the emotional–cognitive–evaluative and sensory–discriminant components of pain in LTMs (Grant, 2014; Grant et al., 2011; Thompson, 2023; Wipplinger et al., 2023). This is reflected in increased pain-related activation in ACC, thalamus, and insula, and decreased pain-related activation in prefrontal cortices, amygdala, and the hypothalamus in LTMs compared with matched naïve controls, with more meditation experience correlating with reductions in these areas and the most significant reductions being displayed in prefrontal regions (dlPFC and the mPFC/orbitofrontal cortex (OFC; Grant et al., 2011)). Additionally, earlier research found greater gray matter in pain-related regions (right dACC and bilateral S2) in LTMs compared with naïve controls. Significant correlations were observed between the number of years of meditation and gray matter thickness in the ACC, as well as between meditation hours and thickness in the lower leg area and right-hand areas of S1 (Grant et al., 2010). These findings suggest a generally increased somatosensory perception (S1, S2) and an enhanced capacity to process affective pain components. Despite also finding significant negative associations between pain sensitivity and cortical thickness of various pain-related regions (right dACC, S2, insula, hippocampus), no interaction effect with the groups was observed, suggesting a general relationship between cortical thickness and pain sensitivity (Grant et al., 2010). Brain activation correlations indicate a decoupling of executive and pain-related brain regions during heat-induced pain, specifically between the right dlPFC and dACC, which strongly predicted reduced pain sensitivity in LTMs (Grant et al., 2011). The enhancement in the sensory–discriminative aspects of pain processing has been suggested not to signify heightened pain perception, but rather an enhanced capability to identify potentially threatening, salient bodily stimuli (Legrain et al., 2011). Nevertheless, the cognitive/sensory information integration component of pain may still be heightened in meditators, with a selective decoupling of emotional appraisal, suggesting an increased differential integration of sensory/cognitive and affective pain components, potentially leading to a heightened but altered pain perception.

Converging results correlated reduced pain unpleasantness in LTMs with enhanced activation in the SN, specifically the dorsal anterior insula and anterior mid-cingulate cortex (aMCC), during the processing of painful heat stimuli. Baseline testing showed the opposite pattern, with decreased SN and amygdala activation, potentially demonstrating reduced pain anticipation and negative affect and increased acceptance and openness during painful sensory stimuli (Lutz et al., 2013). Meditation hours were significantly negatively associated with baseline left anterior insula activation, even after controlling for age. This unique neural signature was also associated with reduced activation over time in the amygdala and pain-processing areas before pain onset, demonstrating a decreased temporal slope of brain activation in the posterior and mid-insula, S2, and mid-cingulate cortex (MCC) in LTMs compared with novices. A similar pattern was observed during pain episodes in the posterior and mid-insula and S2 (Lutz et al., 2013).

Amygdala hyperactivation is evidenced to play a major role in pain processing, particularly regarding the emotional-affective component of pain (Veinante et al., 2013). This key region receives bottom-up sensory pain information from the brainstem as well as polymodal information from the thalamus (Veinante et al., 2013). Its activation is additionally inhibited by mPFC (Neugebauer, 2015). Pathological overactivation of the amygdala and decreased afferent inhibition from mPFC are linked to pain-related disorders, notably physiological pain in chronic pain and emotional pain in MDD patients (Thompson & Neugebauer, 2017). Results are in line with an advanced implicit reappraisal of painful stimuli in the context of long-term meditation, suggesting overall improved self-regulation of pain by attenuating its emotional impact rather than its sensory intensity. This indicates that meditators may have learned to decouple the monitoring of aversive stimulation from the processes that lead to it being labeled or experienced as pain.

To specifically assess the long-term effects of meditation on pain perception, Grant et al. (2010) assembled a cohort comprising 19 long-term Zen meditators and 20 age- and gender-matched controls. The meditators averaged 6,404 hours of mindfulness meditation experience, ranging from 1,229 to 45,000 hours. Employing computer-controlled thermal stimulation, the researchers induced a moderate pain level, prompting both groups to convey their discomfort on a 10-point pain rating scale. The behavioral outcomes revealed a significant decrease in pain sensitivity among Zen meditators, as indicated by a substantial 2-degree Celsius difference during the hot stimulation for the same pain magnitude. These results find support in previous and subsequent studies employing similar research methodologies, consistently observing reduced baseline pain sensitivity and analgesic effects in Zen meditators (Grant & Rainville, 2009; Grant et al., 2011). Notably, in their earlier research, a *focus-and-observe* practice heightened pain sensations among control participants but had no impact on LTMs. Conversely, an *observe-and-release* practice diminished pain in the Zen group without affecting the control group (Grant & Rainville, 2009). In their earlier work, meditation hours successfully predicted reductions in pain sensitivity (Grant & Rainville, 2009). However, this effect was not replicated in their subsequent study (Grant et al., 2010), despite half of the sample being the same. It is still unclear whether there were additional sample overlaps in their most recent study (Grant et al., 2011).

To investigate the effects of different meditation practices on pain, Perlman et al. (2010) studied 9 Tibetan Buddhist LTMs (range: 10,000–45,000 practice hours) and 10 novices. Participants completed eight blocks of four trials of focused attention (FA) or open presence (OP) meditation. Open presence (OP) is a non-dual and advanced form of open monitoring (OM), and, thus, considered an *awareness-of-awareness* meditation. Whereas in OM, the monitoring of the present-moment experience may still contain a duality between meditator and content, OP emphasizes a completely transparent mind in which experience is reflexively aware of itself without a reified subject (Josipovic, 2010, 2019, 2021; Perlman et al., 2010). In FA, participants directed attention to a neutral object other than their pain. Each trial involved 45 seconds of meditation followed by a graded thermal pain stimulus, with participants continuing to meditate and providing pain ratings throughout. A significant interaction emerged between practice type and rating type within the groups, revealing a notable decrease in pain sensitivity and unpleasantness among LTMs practicing OP meditation, with the latter being the primary contributing factor.

Applying a comparable research design, the team validated their results by replicating the study in a group of 14 Tibetan Buddhist LTMs (Lutz et al., 2013) with a minimum of 10,000 and an average of 27,000 hours of OP meditation experience. Compared with a matched control group of 14 meditation-naïve individuals, analysis revealed almost identical pain intensities between groups but diminished unpleasantness in the LTMs. Supplementary analysis uncovered an interaction effect between practice and rating type (intensity and unpleasantness) for the experts but not the control group, showing lower unpleasantness in the expert OP than in the FA group. Still, the expert OP generally outperformed the expert FA group by demonstrating both reduced pain intensity and unpleasantness ratings.

Consistent with previous findings, Zorn et al. found identical reductions in unpleasantness but not intensity after an *observe-and-release (*Kagyu) practice in 27 experienced Tibetan Buddhist meditators (average meditation experience = 41,357 hours; Zorn et al., 2020). In this cross-sectional study, the researchers implemented a design aimed at enhancing cognitive–affective aspects of pain by modulating pain anticipation and stimulus duration. Results indicated a significant decrease in pain catastrophizing in LTMs compared with the novices, which predicted lower sensory–affective connectivity during extended pain stimuli and decreased pain intensity during brief exposures. Subsequent analyses unveiled heightened cognitive defusion—an experiential distancing from thoughts and emotions—as a central psychological mechanism driving the pain-reducing effects of meditation (Zorn et al., 2021). This finding corroborates a reduced negative cognitive–affective pain appraisal in LTMs compared with beginners.

In summary, preliminary evidence suggests that LTMs engaging in *observe-and-release and awareness-of-awareness* techniques consistently demonstrate altered pain perception, specifically diminished pain unpleasantness and, in some instances, intensity (Grant & Rainville, 2009; Grant et al., 2010, 2011; Lutz et al., 2013; Perlman et al., 2010; Zorn et al., 2020). These changes are linked to cognitive decentering from the painful stimulus, resulting in a separation between sensory and emotional components of the pain experience. Conversely to popular models of cognitive control, LTMs’ improvements in pain relief are not based on increased coupling between executive and pain-processing areas (Grant et al., 2011) but instead rely on enhanced bottom-up functional integration in key nodes shared between the SN and pain areas, such as the dACC, anterior insula, and amygdala, regions involved in attentional control and monitoring of salient stimuli (Bush, 2011; Grant, 2014; Lutz et al., 2013; Seeley et al., 2007; Sezer et al., 2022). This suggests that LTMs exhibit enhanced integration of sensory and cognitive aspects of pain, allowing for more precise identification of painful stimuli while reducing negative cognitive valuation and emotional reactivity. The following section reviews eight studies on emotional processing to expand on these Gestalt changes.

### 3.2 Affective processing and long-term meditators

In meditation research, attention-based practices are predominant; however, there is growing interest in other practices, including loving-kindness meditation (*mettā*), due to their potential to profoundly influence emotional subsystems. Indeed, emotion regulation, both through reappraisal and extinction, has been proposed as a central mechanism in meditation (Hölzel et al., 2011; Wheeler et al., 2017). Emotion regulation encompasses conscious and unconscious alterations of emotions through processes that occur before and during the emotion itself (Gross, 1998). For example, attentional deployment on a given salience landscape influences the onset of emotional responses (Gross & Thompson, 2007).

Neuroanatomically, meditation’s effects on emotion regulation have been associated with activation in mPFC, limbic regions, and striatum (Hölzel et al., 2011; Tang et al., 2015), increased coupling between the dorsomedial prefrontal cortex (dmPFC) and rostral anterior cingulate cortex (rACC), and decreased connectivity between amygdala and rACC (Sezer et al., 2022). Research on the neural correlates of trait mindfulness demonstrated a potential association with reduced amygdala activation and ambiguous findings regarding the impact of trait mindfulness on bottom-up and top-down emotion regulation mechanisms (Treves et al., 2024a).

Utilizing a pre- to post-meditation quasi-experimental design, 12 long-term Canadian Zen meditators and 10 age- and sex-matched beginner meditators’ responses to emotional pictures were compared (Taylor et al., 2011). The meditators averaged 1,709 hours of practice experience, spanning between 1,000 and 3,000 hours. Analyses revealed reduced emotional intensity ratings in the meditation condition for negative, neutral, and positive pictures. No differences were found between the meditators and the controls, suggesting a state-dependent effect.

In a similar research design, emotional word evaluation and implicit affective processing were investigated using a lexical decision and valence rating task (Lusnig et al., 2020). The study included 20 German Zen meditators, averaging 1,900 practice hours, and a matched control group. Corroborating the previous results, this study found reduced valence ratings to low-arousal positive and low- and high-arousal negative words in the meditators. However, no differences were found in the time-constrained lexical decision task, suggesting no influence of meditation on automatic emotional word associations. Importantly, meditators meditated before the tasks, while controls watched a movie, complicating the distinction between short-term and long-term effects.

Self-related emotional processing was assessed in 22 long-term *vipassanā* meditators and 22 matched naïve controls using a self-referential word presentation task. During this task, blocks of adjectives—self-praising, self-critical, negative but not self-critical, and neutral—were presented, and affect was assessed after each block (Lutz et al., 2016). The meditators averaged 5,971 total meditation hours, 4,861.5 hours of which were *vipassanā* practice, and including at least 1 retreat. Questionnaire results revealed a trend for enhanced self-compassion and reduced alexithymia (or “emotional blindness”) scores, while demonstrating significantly increased non-activation scores among the meditators. Behaviorally, although overall affective ratings were not significantly predicted by group, the interaction between group and condition was significant. Specifically, the difference in affective ratings between self-related positive and self-related negative adjectives was smaller in LTMs than in meditation-naïve participants. This suggests that LTMs may exhibit more balanced affective responses to self-related stimuli, indicating enhanced emotional regulation or equanimity.

To examine emotional sensitivity, a group of long-term Sant Mat meditators was assessed in their performance in a color perception task when presented with emotional pictures (Chen et al., 2018). Sant Mat meditation is centered on loving-kindness meditation, with a core focus on practicing unwavering compassion. Experts (*n* = 21) meditated on average 4 hours per day over the last 13 years, whereas the controls (*n* = 20) had just started their practice. Behavioral results showed diminished trait and state anxiety in the LTMs, which correlated positively with years of practice. Furthermore, LTMs’ longer reaction times and attenuated accuracy reductions during the explicit presentation of emotional pictures indicated lower hypervigilance and greater neutrality toward explicit emotional stimuli.

The direct effect of loving-kindness meditation (LKM) on affect was studied in 15 LTMs from the Tibetan Nyingma tradition, which is known for its emphasis on cultivating altruism, loving-kindness, and compassion (Engen & Singer, 2015). Averaging 40,000 practice hours, each practitioner completed a compassion meditation condition and a reappraisal control condition. The behavioral outcomes showed that compassion practice increased positive affect and reduced negative affect more than all other conditions, while reappraisal was more effective at reducing negative affect but less so at enhancing positive affect. Both practices outperformed the passive condition in emotional regulation.

Emotion regulation and its associated neural patterns may be pivotal in understanding other areas, such as pain perception, decision-making, and their relationship with foundational cognitive functions. For example, meditators exhibit increased blood-oxygen-level-dependent (BOLD) activation within the insula, particularly within the right posterior region, while the left anterior insula activation decreases when faced with an unfair decision (Kirk et al., 2011). This neural activation pattern suggests a leaning toward greater reliance on interoceptive rather than affective processing during the meditators’ decision-making, as right insula activation has been associated with increased internal attention (Craig, 2003; Hölzel et al., 2008) and momentary self-reference (Farb et al., 2007) in meditators. Positioned as a pivotal hub within the limbic network, the insula plays a central role in regulating emotions and empathy (Hölzel et al., 2011). Specifically, the right insula is associated with perceptual aspects of empathy, potentially reflecting its relevance for body awareness, while the left insula is linked to both perceptual and cognitive–evaluative facets of empathy (Fan et al., 2011).

Consistent with these findings, Tibetan LTMs (range: 10,000–50,000 meditation hours) showed increased right insular BOLD activation in response to negative compared with neutral and positive auditory stimuli during compassion meditation (Lutz et al., 2008a). Notably, deeper meditative states increased insula activity in both groups compared with more superficial blocks. Analysis of interaction effects between meditation and rest revealed that in response to all sound types, experts exhibited increased activation in the right TPJ, right posterior superior temporal sulcus (pSTS), amygdala, mPFC, PCC/Precuneus (PCC/Prc), and inferior frontal gyrus (IFG). Expertise-related effects were especially pronounced in PCC/Prc and right-lateralized pSTS/TPJ regions. This pattern suggests that, compared with novices, experts may display increased social cognition, emotion sharing, and perspective taking (right pSTS/TPJ; Saxe, 2006; Sommerville & Decety, 2006; Tankersley et al., 2007), as well as increased recognition of salient emotional stimuli (right IFG/TPJ; Corbetta & Shulman, 2002) when presented with emotional human vocalizations.

Investigating meditations’ effect on amygdala reactivity, LTMs evidenced increased BOLD activation to explicit happiness, while beginners exhibited the opposite pattern with fear (Chen et al., 2018). LTMs also displayed reduced amygdala activation to implicit emotional states, regardless of valence, and amygdala activation to explicit fear-mediated meditative effects on anxiety. In contrast, reduced amygdala activation was found in both the MBSR program group and the long-term mindfulness meditation cohort to positive images (Kral et al., 2018; see Table 1 for details). Furthermore, only extended practice was associated with reduced amygdala activation to negative stimuli in the latter study, indicating differing patterns of amygdala responses between the two investigations. Specifically, retreat hours involving *observe-and-release* practices were associated with decreased activation to negative images. The increased connectivity observed between the ventromedial prefrontal cortex (vmPFC) and the amygdala within the MBSR group, but not among the experts, suggests that higher-level control mechanisms guide initial adjustments in emotional processing. Over time and with sustained practice, these mechanisms gradually recede as emotional processing becomes more automatic, mimicking mindfulness experience-dependent changes in pain (Cooper et al., 2022; Grant, 2014; Hölzel et al., 2011; Kral et al., 2018; Tang et al., 2015; Wipplinger et al., 2023).

**Table 1.** Biobehavioral cognitive studies of meditation.

Article	Study design	Assessment	Participants	Meditation experience	Meditation type	Findings
Perception						
Fox et al., 2012	Quasi-experimental cross-sectional design (practitioners)	Objective Psychophysical and Cortical Measures of Tactile Sensitivity, Subjective Measure of Sensitivity	Practitioners (N = 38, F = 19)	2,051 hours, SD = 3,600, 1 – 15,000 hours; 11.0 years, SD = 10.3 years. body-scan meditation: 154 hours, SD = 322, 0 – 1,643 hours	Vipassana, body-scan meditation	Experts Introspective accuracy: ↗ MED: r = .31 – .46 ↗ body-scan meditation: r = .41 – .64 Beginners Introspective accuracy: ↘ MED: r = –.01 – –.16 ↗ body-scan meditation: r = .06 – .18 General Expertise (hours) predicting intro. accuracy: ↗ MED: r = .37 – .48 ↗ body-scan meditation: r = .32 – .36 ↗ MED controlled for body-scan meditation: r = .22 – .36
Khalsa et al., 2020	Double-blind RCT (naïve vs. experienced)	Body Map by Drawing, EKG, Self-Report	Meditation-naïve (n = 15, F = 5), Experts (n = 15, F = 5)	4,947 hours, SD = 6,251; 10.8 years, SD = 10.8; Retreat: 19 days, SD = 14	Vipassana, Kundalini	Between-group differences Cardiac interoceptive awareness: No significant differences found.
Grant et al., 2010	Quasi-experimental repeated-measures design (naïve vs. experienced)	Computer-Controlled Thermal Pain Sensitivity Test	Meditation-naïve (n = 20, F = 5), Experts (n = 19, F = 4)	14.4 years, SD = 8.39, 2 – 30 years; 6,404 hours, SD = 8,522, 1,229 – 45,000 hours	Zen: trained in mindfulness meditation	Experts Pain sensitivity: ↘ moderate pain stimulus: p = .002 Expertise predicting cortical thickness: ↗ meditation hours–S1: r = .69, p < .05 ↗ meditation years–ACC: r = .59, p < .05
Grant & Rainville, 2009	Quasi-experimental repeated-measures design, counterbalanced (naïve vs. experienced)	Computer-Controlled Thermal Pain Sensitivity Test	Meditation-naïve (n = 13, F = 5), Experts (n = 13, F = 5)	6,247 hours, SD = 11,789	Zen: trained in mindfulness meditation	Experts Pain sensitivity: ↘ moderate pain stimulus: p = .01 ↘ observing and release: p = .02 Controls Pain sensitivity: ↗ focused observing: p < .001 General Correlations with pain sensitivity: ↘ meditation experience: r = –.82, p < .01
Grant et al., 2011	Quasi-experimental repeated-measures design (naïve vs. experienced)	Computer-Controlled Thermal Pain Sensitivity Test, fMRI	Meditation-naïve (n = 13, F = 4), Experts (n = 13, F = 4)	Not reported	Zen	Experts Pain sensitivity: ↘ moderate pain stimulus: p = .01 Brain activation group differences: ↘ dlPFC, mPFC/OFC, amygdala, hippocampus, IFG, MFG ↗ dACC, insula, thalamusBrain activation predicting pain sensitivity: ↘ dACC-dlPFC connectivity: r = –.71, p < .001 Brain activation correlating with meditation experience: ↘ years – bilateral insula: r = -.60 – –.65, p < .05 ↘ years – bilateral dACC: r = –.69, p < .01 ↘ years–left dlPFC: r = –.80, p < .001 ↘ right mPFC/OFC: r = –.64, p < .05 ↘ hours–thalamus: r = –.56 – –.62, p < .05
Perlman et al., 2010	Quasi-experimental repeated-measures design, counterbalanced (naïve vs. experienced)	Computer-Controlled Thermal Pain Sensitivity Test	Meditation-naïve (n = 10, F = 5), Experts (n = 9, F = 4)	10,000–45,000 hours	Tibetan Buddhism: Kagyu and Nyingma	Experts Pain sensitivity: ↘ observing and release: p = .011 Pain unpleasantness: ↘ observing and release: p = .013
Lutz et al., 2013	Quasi-experimental repeated-measures design, counterbalanced (naïve vs. experienced)	Computer-Controlled Thermal Pain Sensitivity Test, fMRI	Meditation-naïve (n = 14, F = 9), Experts (n = 14, F = 9)	27,000 hours, SD = 12,500	Tibetan Buddhism: Kagyu and Nyingma	Experts Pain unpleasantness: ↘ p = .001 ↘ observing and release: p < .001 Brain activation baseline group-differences: ↗ dorsal anterior insula, aMCC, amygdalaBrain activation during pain group differences: ↗ dorsal anterior insula, aMCCBaseline brain activation correlating with meditation experience: ↗ left anterior insula: r = –.63, p < .05 Neural habituation before pain stimulus: ↗ amygdala, right posterior insula, right S2, right mid-insula, MCCNeural habituation before pain stimulus: ↗ right posterior insula, right S2, right mid-insula
Zorn et al., 2020	Quasi-experimental repeated-measures design, counterbalanced (beginner vs. experienced)	Computer-Controlled Thermal Pain Sensitivity Test, Pain Catastrophizing Scale	Meditation beginner (n = 14, F = 9), Experts (n = 14, F = 9)	Experts: 41,357 hours, SD = 17,999, 13,110–94,535 hoursBeginners: 19.4 hours, SD = 12.9, 2.2–49.2 hours	Tibetan Buddhism: Kagyu and Nyingma	Pain unpleasantness Experts: ↘ observing and release: p = .021Beginner: ↘ observing and release: p = .018
Emotional processing						
Taylor et al., 2011	Quasi-experimental repeated-measures design (beginner vs. experienced)	Emotional Intensity Ratings	Meditation beginner (n = 10, F = 4), Practitioners (n = 12, F = 7)	1,709 hours, SD = 694, 1,000–3,000 hours, excluding 1 outlier of 45,000 hours	Zen	General Emotional intensity: ↘ all pictures, meditative state: p < .05 Experts Brain activation: ↘ right mPFC: p < .005 ↘ right PCC: p < .005 Beginner Brain activation: ↘ left amygdala: p < .005
Lusnig et al., 2020	Quasi-experimental repeated-measures design (naïve vs. experienced)	Lexical Decision Task, Valence Rating Task	Mediation naïve (n = 20, F = 9), Practitioners (n = 20, F = 9)	7.9 years, SD = 4.9, 0.5 – 28 years; 1,900 hours	Zen	Mediators Valence ratings post-mediation: ↘ positive, low arousal: p < .001 ↘ negative, low arousal: p < .001 ↘ negative, high arousal: p < .001
Chen et al., 2018	Quasi-experimental repeated-measures design, counterbalanced (naïve vs. experienced)	State and Trait Anxiety, Color Identification Task, Emotion Detection Task, fMRI	Mediation naïve (n = 20, F = 12), Experts (n = 21, F = 14)	12.95 years, SD = 6.1, 4 – 26 years; 4 hours daily	Sant Mat: Loving-kindness meditation	Mediators State and trait anxiety: ↘ state: p < .001 ↘ trait: p = .001 ↘ state anxiety correlating with mediation experience: r = –.48, p = .001 ↘ trait anxiety correlating with mediation experience: r = –.48, p = .001Color Identification Task: ↗ RT independent of attention and emotion: p = .001 ↗ RT, explicit: p < .05 ↗ accuracy, explicit: p < .05 Brain activation, explicit happy: ↗ Amygdala: p < .005 Amygdala activation mediating reductions in Anxiety: ↘ fear, explicit: p < .05 Controls Brain activation, explicit fearful: ↗ Amygdala: p < .005 Brain activation, implicit fearful: ↗ Amygdala: p < .005 Brain activation, implicit happy: ↗ Amygdala: p < .005
Engen & Singer, 2015	Quasi-experimental repeated-measures crossover design, counterbalanced (experienced)	Visual Analogue Rating of Affect, Emotion Regulation Task, fMRI	Experts (N = 15, F = 5)	40,000 hours, SD = 9,000, 10,000–62,000 hours	Tibetan Buddhism: Nyingma tradition	Compassion meditation Positive affect: ↗ compared with all other conditions: p < .01 Negative affect: ↘ compared with watch negative: p < .001 Brain activation, before stimulus: ↗ mOFC: p < .001 ↗ VS/NACC: p < .001 ↗ bilateral mid-insula: p < .001 Reappraisal condition Positive affect: ↗ compared with watch negative: p < .01 Negative affect: ↘ compared with watch negative: p < .001 ↘ compared with compassion: p < .01
Lutz et al., 2008a	Quasi-experimental repeated-measures design (naïve vs. experienced)	Human Emotional Vocalizations, fMRI	Mediation naïve (n = 15, F = 2), Experts (n = 15, F = 2)	10,000–50,000 hours	Tibetan Buddhism: Nyingmapa and Kagyupa	Experts Negative sounds vs. others while meditating: ↗ right insula: p < .05 Good vs. poor mediation blocks: ↗ right insula: p < .05 Mediation vs. rest: ↗ right TPJ: p < .001 ↗ right pSTS: p < .001 ↗ Amygdala: p < .001 ↗ PCC/Prc: p < .001 ↗ IFG: p < .001 ↗ mPFC: p < .001
Kral et al., 2018	Quasi-experimental, cross-sectional longitudinal comparison (experienced vs. vs. MBSR)	Automatic Emotion Regulation Task, fMRI	MBSR (n = 32, F = 22), Experts (n = 30, F = 16)	9,081 hours, 1,439–32,612 hours	Mindfulness meditation, some Loving-kindness meditation	Experts Brain activation: ↗ right amygdala, positive: p = .001 Lifetime retreat hours–observing and release: ↘ Amygdala, negative: p = .02 MBSR Brain activation: ↗ right amygdala, positive: p = .01 ↗ amygdala–vmPFC connectivity, positive: p = .01 ↗ amygdala–vmPFC connectivity, negative: p = .001
Lutz et al., 2016	Quasi-experimental repeated-measures design (naïve vs. experienced)	Self-referential Word Processing Task, Five-Facet Mindfulness Questionnaire, Toronto Alexithymia Scale, Self-Compassion Scale, fMRI	Meditation naïve (n = 22, F = 8), Experts (n = 22, F = 10)	5,971 hours, 506–18,805 hours. Vipassana: 4,861.5 hours, 281–18,325 hours.	Vipassana	Experts Questionnaires: ↗ self-compassion: p < .1 ↘ alexithymia: p < .1 ↗ non-activation: p = .002 Affect: ↘ affective difference between self-praise and self-criticism: p < .05 Brain activation for self-appraisal conditions: ↗ dmPFC ↘ dmPFC-Prc and dmPFC-occipital connectivity, positive Psychophysiology for self-appraisal conditions: ↗ dmPFC correlating with non-activation: r =.49, p = .03
Non-Ordinary States of Consciousness						
Berkovich-Ohana et al., 2011	Quasi-experimental repeated measures design (naïve vs. experienced)	Time Production Task	Meditation naïve (n = 12) vs. Mindfulness meditators (n = 36, 12 per group); Meditation-naïve (n = 9) vs. TM (n = 10)	ST MM (894 hours, SD = 450), IT MM (2,570 hours, SD = 471), LT MM (7,556 hours, SD = 502), TM (16,310 hours, SD = 11,970)	Mindfulness meditation, Transcendental meditation	Mindfulness meditators Time duration: ↗ compared with control, premed.: p < .005 ↗ compared with control, postmed.: p < .005
Berkovich-Ohana et al., 2013	Quasi-experimental repeated measures design (experienced)	Specific Meditation Task, MEG, Phenomenological Analysis	Experts (N = 12, F = 3)	16.5 years, SD = 7.9; 9 – 34 years; 11,225 hours, SD = 9,909, 1,290–29,290 hours	Theravada Buddhism: mindfulness meditation	Experts Brain activation, Spacelessness/ Timelessness: ↗ PCC: p < .05 ↗ right TPJ: p < .05 ↗ cerebellum: p < .05
Gutiérrez et al., 2022	Quasi-experimental repeated measures design, counterbalanced (experienced)	Self-Report Questionnaires, Metronome Task	Experts (N = 22, F = 12)	19.2 years, SD = 14.83; 2,994 hours, SD = 3,213; 4.0 hours weekly, SD = 2.08	Mindfulness, Vipassana, Zen, Tibetan Buddhism	Experts Body-boundaries: ↘ during meditation: p = .003 Time-passage: ↗ during meditation: p = .014 Attention to time: ↘ during meditation: p = .003
Nave et al., 2021	Quasi-experimental repeated measures design, counterbalanced (experienced)	Interviews, State and Trait Anxiety Questionnaire, 5 Dimensions-Altered States of Consciousness Questionnaire, Dissociative Experience Questionnaire	Experts (N = 49, F = 19)	3,832 hours, SD = 4,845, 115–24,837 hours	Theravada Buddhism	Experts Degree of dissolution: ↗ observing and release: p < .001 ↗ correlation with lifetime meditation hours: r = .52, p < .001 First-person perspective: ↗ correlation with lifetime meditation hours: r = .49, p < .01 Attention: ↗ correlation with lifetime meditation hours: r = .44, p < .01 Agency: ↗ correlation with lifetime meditation hours: r = .65, p < .001
van Lutterveld et al., 2017	Quasi-experimental repeated measures (beginner vs. experienced)	Effortless Awareness Neurofeedback Task, EEG	Meditation beginner (n = 16, F = 5), Practitioners (n = 16, F = 6)	6,164 hours, 1,527–50,978 hours	Heterogenous, mostly Theravada/Vajrayana	General Effortless awareness: ↘ PCC activation: p < .0025, extended to moment-to-moment correspondenceVolitional PCC control: ↗ effortless awareness meditation: p < .0005
Decision-making						
Kirk et al., 2011	Quasi-experimental repeated measures design (beginner vs. experienced)	Post-Ultimatum Game Interview, fMRI	Meditation-naïve (n = 40, F = 21), Experts (n = 26, F = 10)	9.5 years, SD = 7.8	Buddhist meditation	Experts Behavioral: ↗ asymmetric offers: p < .01 Brain activation: ↗ posterior insula, unfair: p < .001 ↘ anterior insula, unfair: p < .001 ↗ left PCG, rational: p < .001 ↗ left pSTC, rational: p < .001 ↗ bilateral PHG, rational: p < .001 Controls Brain activation: ↗ anterior insula, unfair: p < .001 ↗ bilateral dlPFC, rational: p < .001
van Vugt & van den Hurk, 2017	Quasi-experimental,cross-sectional longitudinal comparison (experienced vs. mediation naïve vs. MBSR vs. active control)	Attentional Network Task, Drift Diffusion Model	Meditation naïve (n = 24, F = 17), Practitioners (n = 24, F = 17)	0 – 27 years	Vipassana	Meditators Decision threshold: ↗ compared with controls: p < .05 ↗ incongruent: p < .001 General Decision threshold: ↗ incongruent trials: p < .001 ↘ main effect of cues: p < .001 Drift rate: ↘ incongruent trials: p < .001 Drift variability: ↗ time-space cue: p < .05 ↘ incongruent trials: p < .05

Study design, assessment type, practice experience, meditation type, and summary of major findings of each article. N = number of participants.

Note. ACC: anterior cingulate cortex; aMCC: anterior midcingulate cortex; dACC: dorsal anterior cingulate cortex; MFG: middle frontal gyrus; PCG: postcentral gyrus; PHG: parahippocampal gyrus; pSTC: posterior superior temporal cortex; SFG: superior frontal gyrus; dlPFC: dorsolateral prefrontal cortex; IFG: inferior frontal gyrus; MCC: midcingulate cortex; mOFC: medial orbitofrontal cortex; mPFC: medial prefrontal cortex; PCC: posterior cingulate cortex; PCC/Prc: posterior cingulate cortex/precuneus; S1: primary somatosensory cortex; S2: secondary somatosensory cortex; VS/NACC: ventral striatum/nucleus accumbens; TPJ: temporoparietal junction.

Other neural research is more ambiguous. For instance, Chen et al. (2018) found increased functional connectivity between amygdala and ventrolateral PFC for happiness, and reduced connectivity between insula and medial orbitofrontal cortex (mOFC) for fear, indicating emotion-dependent corticolimbic connectivity changes in meditators. Another study did not identify a link between improved emotion regulation and cognitive control mechanisms; instead, it suggested a connection to maintaining acceptance and presence during emotional states (Taylor et al., 2011).

Conversely, increased BOLD activation of frontal and limbic regions was observed during emotional self-appraisal in long-term *vipassanā* practitioners, most strongly for positive appraisal (Lutz et al., 2016). Specifically, enhanced dmPFC activation was associated with self-reported reductions in habitual emotional non-activation. Taylor et al.’s Zen meditators exhibited reduced BOLD activation in both frontal and posterior key DMN regions, particularly the right PCC and right mPFC, compared with attenuated activation in the left amygdala among their novice participants. As the DMN is associated with self-referential processing, these findings suggest that LTMs’ improved emotion regulation may be due to altered self-awareness instead of enhanced cortico-limbic control. Indeed, despite observing increased BOLD activation in the dmPFC, Lutz et al. (2016) demonstrated reduced functional connectivity between the dmPFC and posterior midline nodes (PRc). This aligns with the literature suggesting an association between pre-reflective self-awareness and dmPFC activity (Farb et al., 2007) as well as reduced within-DMN connectivity (Cooper et al., 2022; Treves et al., 2024a).

To investigate the neural effects of meditation practices and expertise on affective processing, FA and LKM practitioners with varying degrees of expertise were recruited (Lee et al., 2012). Long-term FA meditators exhibited heightened BOLD activation in the left insula when exposed to happy pictures during meditation, whereas the long-term LKM group showed increased activation in left ventral ACC, right IFG, and right Prc. When confronted with sad pictures during meditation, the long-term FA group displayed significant left SFG and right IFG activation, while the long-term LKM group showed middle frontal gyrus (MFG) and left caudate activation.

These areas have been associated with reorienting attention to endogenous stimuli (MFG; Japee et al., 2015), working memory (SFG; du Boisgueheneuc et al., 2006), attentional control and response inhibition (IFG; Swick et al., 2008; Hampshire et al., 2010; Liakakis et al., 2011), and several other higher-order functions, such as speech, language comprehension, reasoning, and empathy (Li et al., 2013; Liakakis et al., 2011). Consequently, these findings suggest enhanced activation in attentional and emotional regions (insula, SFG, IFG) among FA experts, irrespective of picture valence. Conversely, LKM experts exhibited increased activation in brain regions associated with emotion identification (left ventral ACC), regulation (right IFG), and self-referential processing (Prc) when viewing happy pictures, and heightened activation in regions (left MFG/caudate) linked to emotion activation and voluntary emotion regulation when viewing sad pictures.

These results suggest that LKM practice may facilitate greater emotional sharing and enhance the ability to cultivate positive emotions in response to affective stimuli. Increases in positive affect may not simply reflect reactions to external stimuli, but rather indicate an internally generated positive emotional and motivational state. Indeed, Engen and Singer (2015) demonstrated a heightened activation of affiliation, positive affect, and reward processing centers, including the bilateral mid-insula, ventral striatum/nucleus accumbens (VS/NACC), and mOFC, before stimulus onset, potentially suggesting an increase in baseline positive affect in LTMs engaging in compassion practice. LKM/compassion practice may thus be particularly helpful in interpersonal situations in which emotional connection predominates over the mere regulation of affective reactions to stressful situations.

In conclusion, neuroimaging of LTMs reveals significant alterations in cortical, limbic, and basal ganglia regions associated with emotional processing, attentional and cognitive control, self-awareness, social cognition and empathy, reward and motivation, interoception, and language and speech processing. The precise neuroanatomical configuration underlying these changes remains to be investigated, as heterogeneity in tasks, meditative experience, and meditative activation prevented homogeneous neural patterns from emerging. Previous research suggested practice- and experience-dependent differences in emotion regulation, with LTMs potentially relying on bottom-up and beginners on top-down regulation strategies (Chiesa et al., 2013). Our findings partially corroborate this view (Kral et al., 2018; Taylor et al., 2011) but also suggest a more complex picture, where frontal coupling with limbic structures may be both increased or decreased (Chen et al., 2018) depending on the task and/or meditation employed. Increased corticolimbic coupling in LTMs demonstrated different signatures, such as connections between more medial (i.e., mOFC) and subcortical structures (Engen & Singer, 2015), indicating that emotion regulation in this population may depend on a unique mix of bottom-up and top-down strategies (Lutz et al., 2008a, 2016). The observed behavioral improvements may, like reductions in pain, depend on greater equanimity, reflected in enhanced emotional neutrality, acceptance, reduced judgment, and negative affect, while compassion meditation may elevate positive affect, social cognition, and mentation. Additional research is needed to clarify how diverse meditation practices affect emotional processing across meditative development.

### 3.3 Decision-making—higher-order processes in long-term meditators

Higher-order cognition refers to advanced processes beyond basic perceptual, sensory, and memory processing. These involve higher-level mental operations such as abstraction, analysis, synthesis, evaluation, planning, and cognitive flexibility, enabling individuals to perform complex problem-solving, reasoning, and decision-making (Luna et al., 2015; Miller & Cohen, 2001; Miller & Wallis, 2009; Unsworth et al., 2009). Here, we review the two studies on decision-making in LTMs.

Previous research has demonstrated improvements in both social and non-social decision-making among meditators, likely due to enhanced emotion regulation, empathy, and cognitive control (Sun et al., 2015). Decision-making involves selecting options based on evaluating costs and benefits. Leading theories, such as the “emotion-imbued choice model,” suggest that emotional processes directly and indirectly influence decision-making, combining both rational and non-rational elements (Lerner et al., 2015). Other factors, such as cognitive biases, heuristics, information constraints, time limitations, and social influences, also shape decisions, with non-rational choices often upholding social conventions (Boyd et al., 2003).

Key prefrontal regions (dlPFC, ACC, mOFC) are crucial for decision-making (Wallis, 2007), while subcortical (insula, amygdala) and other cortical areas (TPJ, vmPFC, dmPFC, IFG, pSTS) support social decisions (Sun et al., 2015). Cortical areas are associated with long-term outcomes, while subcortical are associated with short-term outcomes, reflecting a recursive top-down model (Rosenbloom et al., 2012).

A quasi-experimental study examined meditation-induced trait changes on decision-making in 26 skilled Buddhist meditators (9.5 years of meditation experience) and 40 non-meditators as controls (Kirk et al., 2011). Both groups’ economic decisions were assessed while playing the Ultimatum game. Participants played 45 rounds of the game as responders, facing human partners for 30 rounds and computer partners for 15 rounds, with predetermined offers varying in fairness. The task assessed their willingness to accept “unfair” offers for prosocial reasons versus rejecting them for fairness. The behavioral results unveiled more rational decision-making in LTMs compared with controls, with the former accepting more than half of the most asymmetrical offers while the latter accepted only a quarter.

In social interactions involving rewards, most individuals tend to evaluate their rewards relative to their peers (Boyd et al., 2003). However, these findings suggest a distinct behavior among meditators, who often accepted even the most unfair offers. Corresponding with the behavioral results, the meditators exhibited increased activation in the posterior insula, an area central to interoceptive processing (see Section 3.2. Emotional Processing). Anterior insula BOLD activation associated with affective processing was decreased in the LTMs, with the opposite observed in the controls. Additional subanalyses showed increased dlPFC activation in particularly rational control participants, whereas meditators exhibited increases in the postcentral gyrus (PCG), posterior superior temporal cortex (pSTC), and parahippocampal gyrus. Consequently, in addition to LTM’s enhanced rational decision-making, rationality may be associated with different neural signatures, with meditators relying on altered somatosensory and perceptual functioning, such as body awareness-induced feeling states (PCG; Critchley et al., 2004; Lutz et al., 2008a), perspective taking (pSTC; Hampton et al., 2008), and altruism (pSTC; Tankersley et al., 2007), and controls on cognitive control (dlPFC; Hare et al., 2009).

To examine how meditation’s attentional manipulation influences decision-making, recent research applied the drift-diffusion model (DDM) (van Vugt & van den Hurk, 2017). The researchers simulated accuracy and response time shifts using the attentional network task across two datasets involving LTMs (For dataset one, see: van den Hurk et al., 2010; dataset two: see Table 1). The attentional network task assesses the efficiency of alerting, orienting, and executive control by measuring reaction times and accuracy in response to visual cues and targets that vary in location and congruence. The DDM provides a computational framework for understanding decision-making by modeling how individuals integrate noisy evidence over time to choose between competing alternatives (Fudenberg et al., 2020). Evidence accumulates toward decision thresholds representing response options (e.g., “left” and “right”). Once a threshold is crossed, the decision is made. In the DDM, the drift rate represents the speed of evidence accumulation, influenced by the ability to extract relevant information from a stimulus. Researchers systematically manipulated cues and congruency to fit these parameters within the DDM.

Group-independent findings revealed a heightened decision threshold, reduced drift rate (information quality), and lower drift variability (sustained attention) during incongruent trials, while informative cues decreased the decision threshold and increased drift rate variability. This aligns with prior research, linking uncertainty to reduced choice readiness and broader information sampling. Additionally, meditators showed higher decision thresholds compared with non-meditators, indicating a more deliberate decision-making process, especially during incongruent trials.

In summary, the findings suggest that LTMs exhibit enhanced rational decision-making with increased decision thresholds, particularly in the face of conflicting information—indicating a tendency to accrue more information and maintain objectivity before committing to a choice.

### 3.4 Non-ordinary states of consciousness and long-term meditators

Non-ordinary states of consciousness (NSCs)—characterized by alterations in time, body, self, and space perception—emerge in both clinical and non-clinical contexts, either spontaneously or induced by meditation, psychedelic substance use, fasting, or hypnosis (Millière et al., 2018; Timmermann et al., 2023; Wright et al., 2024). Neurophenomenology, conceived as a functional solution to the “hard problem of consciousness,” involves merging objective brain data with subjective experience and has become central to exploring various facets of NSCs, including meditation-induced self-boundary dissolution (Berkovich-Ohana et al., 2020), cessations of consciousness (Chowdhury et al., 2023; van Lutterveld et al., 2024), and deep concentrative absorptive states (Chowdhury et al., 2024; Demir et al., 2025; Ganesan et al., 2024; Potash et al., 2024, 2025; Sparby & Sacchet, 2024; Treves et al., 2024b; Yang et al., 2023, 2024a).

Despite their prevalence, NSCs remain vastly understudied (Wright et al., 2024) and thus may particularly benefit from research in advanced meditation and emergent phenomenology to better identify and support individuals experiencing adverse effects from these experiences (Sandilands & Ingram, 2024; Wright et al., 2024). Recent multidimensional frameworks for the study of self-consciousness (Millière et al., 2018) and neurophenomenology (Timmermann et al., 2023) can provide a structure for comprehensively investigating the subtle differences and similarities between various modalities (e.g., meditation and psychedelics) and internal and external contextual factors (e.g., personality and culture).

To explore changes in temporal cognition, researchers compared four meditation groups with age-matched controls using a pre–post meditation design (Berkovich-Ohana et al., 2011). The groups included three mindfulness-based cohorts: short-term (894 hours of meditation experience), intermediate (2,570 hours), and long-term (7,556 hours), as well as a *transcendental meditation* group (16,310 hours), a technique involving the silent repetition of a mantra while maintaining focused awareness. A time production task was employed, where participants pressed a button to match target durations signaled by a sound, with intervals presented in a random order, and their eyes closed. This method measured time estimation accuracy by comparing produced durations to the target durations.

Results revealed significantly increased time production in the mindfulness groups but not in the other groups. Time production was unaffected by meditative state or experience, supporting the cognitive-timer model, which links lower arousal and heightened attention, characteristic of mindfulness meditation, to increased time production. The researchers proposed that mindfulness meditators may rely more on momentary core consciousness based on sensorial rather than memory processing.

Corroborating this finding, a neurophenomenological study using magnetoencephalography (MEG) in 12 Theravada LTMs (mean 11,250 hours of practice) linked experiences of “timelessness” and “spacelessness” to neural changes in bodily processing, evidenced by increased theta-band activity in the right TPJ, PCC/Prc, and cerebellum (Berkovich-Ohana et al., 2013). Theta-band activity is observed in states of deep relaxation (i.e., meditation and hypnosis) and has been associated with temporospatial processing and broad neuropsychological integration (Hasselmo & Stern, 2014; Hunt, 2007; Vaitl et al., 2005). The PCC and Prc have been linked to consciousness (Cavanna & Trimble, 2006), TPJ to first-person multisensory body processing (Decety & Lamm, 2007), and the cerebellum to altered body awareness (Barmack, 2003). These NSCs were distinct from the memory and imagination processes in the control “then” and “there” conditions, in which meditators were instructed to situate themselves in the near past or a specific location rather than in the present or outside of time and space. Notably, neurophenomenologically guided analyses revealed decreased activation in the right TPJ and insula, and increased activation in the cerebellum, among participants who were more capable of inducing NSCs of “timelessness” and “spacelessness.”

Another study recruited 22 LTMs (mean 2,994 practice hours) practicing present-moment techniques common to Zen, *vipassanā*, Tibetan Buddhism, and secularized mindfulness (Gutiérrez et al., 2022). Using a within-subjects design, they compared first-person reports of time perception and self-boundaries before and after 20 minutes of meditation versus a reading control. Meditation led to diminished bodily boundaries, a sense of time elongation, and reduced attention to time compared with the reading condition. To assess present-moment awareness, a metronome task was used to measure the integration of successive beats as a proxy for present-moment duration. Contrary to prior findings, no significant differences emerged between groups, possibly due to participant misunderstanding or trait-based ceiling effects.

These and other findings indicate that changes in perceived time and self often co-occur in NSCs (Droit-Volet & Dambrun, 2019). Investigating one can thus offer valuable insights into the other: understanding alterations in time perception can shed light on changes in self-experience and vice versa.

Exploring self-dissolution, Nave et al. investigated the phenomenological profiles of 46 LTMs from various traditions following a 3-week Theravada meditative training (Nave et al., 2021). Participants averaged 3,832 hours of practice (range: 115–24,837 hours), including at least one retreat. Using microphenomenology and quantitative analysis, six experiential dimensions were assessed: perception of location, agency, first-person perspective, attention, body sensations, and affective valence. The study identified a primary dimension of boundary dissolution, driven by changes in agency and, to a lesser extent, location. Dissolution was linked to more positive affect than maintaining bodily boundaries. *Observe-and-release* practices, which relax attention and agency, were especially effective in promoting dissolution. According to the authors, these findings align with the enactive view of the minimal self, which roots selfhood in foundational sensorimotor and attentional processes. Moreover, total meditation experience correlated weakly to moderately with the degree of dissolution and experiential shifts in attention, first-person perspective, and agency, indicating that greater experience was associated with broader, more dynamic attention, a passive sense of agency, and non-dual awareness.

Indeed, prolonged meditation has been shown to induce “effortless awareness,” a state marked by the absence of focused attention and cognitive effort, and accompanied by perceptual alterations (e.g., of space). To investigate this, researchers examined the effects of targeted electroencephalogram (EEG) neurofeedback during effortless awareness meditation on meditative depth in 16 novice and 16 LTMs (6,164 meditation hours) from various traditions (van Lutterveld et al., 2017). Gamma band activity in the PCC—a DMN hub inversely associated with effortless awareness—was used as neurofeedback. Decreased PCC activation closely corresponded with reports of effortless awareness across both groups, with no significant group differences. These effects were observed moment-to-moment, and participants were able to modulate PCC activity during meditation in real time.

These findings align with research indicating that reduced DMN activity and connectivity, especially of the mPFC and PCC, alongside increased insula activation, may index mindfulness-related changes in self-awareness (Brewer et al., 2011; Hölzel et al., 2011; Millière et al., 2018; Tang et al., 2015; Treves et al., 2024a), fostering a transition from a temporally extended narrative self to a more experiential, dynamic, and de-reified sense of self (Farb et al., 2007; Gallagher et al., 2023; Tang et al., 2015; Timmermann et al., 2023; Vago & Silbersweig, 2012). This is further reflected in a study investigating the synergistic effects of psychedelics on meditation during a Zen retreat, where mPFC and PCC decoupling predicted ego-dissolution (Smigielski et al., 2019) and in a recent longitudinal transcranial-focused ultrasound study, where PCC inhibition increased mindfulness, altered the sense of self, time, and memory, and reduced rsFC within the DMN and between PCC and dlPFC (Lord et al., 2024b).

In sum, LTMs may exhibit an enhanced capacity to modulate their experience of time and space, often accompanied by varying degrees of self-boundary dissolution. These NSCs appear grounded in altered bodily and self-processing, alongside a reduced reliance on memory. Inconsistent findings may stem from experienced but less advanced meditators. This suggests that NSCs-related designs may be particularly well suited to differentiating between these populations. Nonetheless, due to substantial overlap across experiential dimensions, isolating distinct phenomenological patterns remains a significant challenge.

## 4 Discussion, Limitations, and Future Directions

### 4.1 Methodological limitations in the study of long-term meditators

To properly contextualize the results and patterns observed in this review, it is important to examine the methodological quality of the research. Numerous researchers have raised concerns about the state of contemplative neuroscience, citing inconsistent findings and false positives (Kral et al., 2022). These issues are often attributed to the broad use of “mindfulness” as an umbrella term, variability in study design and reporting, and differing levels of methodological rigor (Sezer et al., 2022; Vago et al., 2019; van Dam et al., 2018). Key areas for improvement include enhancing construct validity, increasing ecological validity, and addressing potential adverse effects (Van Dam et al., 2018). As a result, interpretations tend to be highly context dependent, limiting generalizability. Future work will likely require new measurement tools to tease phenomenology from appraisal, refined temporal models of meditative development, larger samples, and greater methodological rigor through preregistration (Galante et al., 2023). Given these complexities, we, in Galante et al. (2023), proposed studying meditation within unified developmental models to advance the understanding of the subject. The following discusses methodological limitations in LTM studies, with the aim of informing steps toward such a unified framework.

One key area concerns predictors of meditation effects, including dose, technique, individual characteristics, phenomenological patterns, and sociocultural context. A major limitation lies in the imprecise reporting of meditation dose and techniques, which stifles interpretations related to LTM’s trait effects. Broad classifications such as “Buddhist meditation” (Kirk et al., 2011) obscure distinctions between practices and their specific cognitive effects. Furthermore, whereas some studies defined mindfulness meditation as an equal balance of *samatha (focused observing)* and *vipassanā (observing-and-releasing*; Kral et al., 2018; van Vugt & van den Hurk, 2017), others did not define the meditative activity at all (i.e., *vipassanā* in Khalsa et al., 2008). Other studies ambiguously referred to Zen practice as based on *observe-and-release* activities (Grant et al., 2011) or mindfulness meditation (Grant & Rainville, 2009; Grant et al., 2010; Taylor et al., 2011) or both, equating mindfulness meditation with *observe-and-release* activities (Lusnig et al., 2020).

To examine trait or state–trait interaction effects, it is crucial to clearly distinguish habitual practices from those used experimentally. Similarly, meditation dose requires systematic quantification: some studies cite only total years of meditation (van den Hurk et al., 2010), others report total meditation hours (Lee et al., 2012), or both (Grant et al., 2010), while several omit mean practice amounts altogether (Lutz et al., 2008a; Perlman et al., 2010; van Vugt & van den Hurk, 2017). Few describe average daily practice amounts before the experiment (Sparby & Sacchet, 2022).

A second methodological issue involves the sociocultural and historical context of the practice. Most studies lacked detailed demographic data, especially concerning race, ethnicity, and cultural background, often reporting only the country of recruitment. This is concerning for multiple reasons. First, cultural background significantly influences cognitive processes by shaping the external representations and tools people use, the content of their thoughts, and their habitual practices, thereby potentially affecting how information is processed, what cognitive strategies may be employed, and even the neurological structures of the brain involved (Bender & Beller, 2013; Kitayama & Murata, 2013; Lao et al., 2013; Nisbett & Miyamoto, 2005). Second, meditative practices are deeply rooted in centuries-old cultural traditions of Buddhist origin (Bodhi, 2011; Nilsson & Kazemi, 2016; Sharf, 2014; Sugunasiri, 2008). Existing research has yielded concerning findings regarding the cross-cultural validity of psychometric measures, for instance, American college students scoring similarly to Theravada monks on mindfulness measures (Christopher et al., 2009). Third, little to no data were collected on advanced phenomenological states, leaving no information on whether meditators’ experience had reached significant stages of meditative development and advanced meditation more broadly. Although several studies reported over 10,000 hours of lifetime meditation experience, the absence of first-person data limited assessments of meditative mastery, making it difficult to distinguish between LTMs and AMs (Galante et al., 2023; Sacchet et al., 2024; Sparby & Sacchet, 2022; Yang et al., 2024a). A comprehensive retrospective phenomenological assessment may still exceed the reach of current contemplative science, as it requires systematic efforts to scientifically evaluate, translate, and map traditional stages of development to contemporary empirically informed models (Grabovac, 2015; Sparby & Sacchet, 2022; Wright et al., 2023). However, such work is essential for bridging behavioral, neural, and phenomenological perspectives, enabling individualized, multivariate models that clarify links between first-person accounts of well-being or psychological distress and cognitive processes.

A third methodological limitation concerns the quality and comparability of neuroimaging data. As given in Table 1, sample sizes in the reviewed studies were small to modest. There was also considerable variability in scan lengths and preprocessing pipelines—factors known to affect the reliability and interpretability of functional connectivity estimates (Nee, 2019; Turner et al., 2018; Parkes et al., 2018; Marek et al., 2022). In addition, substantial individual variability related to meditation predictors likely influenced the robustness of findings. These issues underscore the need for unified research frameworks (Galante et al., 2023; Sparby & Sacchet, 2022; Wright et al., 2023), standardized preprocessing protocols, and open data practices to support robust group comparisons and enable large-scale meta-analytic synthesis (Esteban et al., 2019; Nichols et al., 2017).

In sum, these methodological concerns extend previous critiques of the field (Sezer et al., 2022; Galante et al., 2023; Vago et al., 2019; van Dam et al., 2018) and underscore the need for a unified research framework, particularly for advanced meditation (Sacchet et al., 2024). In the next section, we summarize the behavioral trait effects reviewed and delineate the differences between meditative activities.

### 4.2 Behavioral trait effects

While most studies in this review report substantial cognitive changes in LTMs, methodological variability limits firm conclusions about trait-level effects (see Table 1). We begin by summarizing the main findings before addressing key limitations.

Across studies, LTMs exhibited between-group improvements in interoceptive accuracy (Fox et al., 2012), though not for cardiac interoceptive awareness (Khalsa et al., 2020), reductions in pain sensitivity (Grant & Rainville, 2009; Grant et al., 2010, 2011; Perlman et al., 2010) and unpleasantness (Lutz et al., 2013; Perlman et al., 2010; Zorn et al., 2021). Emotional outcomes included increased neutrality (Chen et al., 2018; Engen & Singer, 2015; Lusnig et al., 2020), positive affect (Engen & Singer, 2015), and reduced negative affect (Chen et al., 2018). Concomitant changes in emotional neutrality and affect may be related to the study design, as well as the cognitive tasks and measurements employed, rather than differences in meditative activities. Meditation also reliably induced increased time production (Berkovich-Ohana et al., 2011), an elongated sense of time passing (Gutiérrez et al., 2022), and states of effortless awareness (van Lutterveld et al., 2017), but without significant group differences, suggesting state-dependent rather than trait effects. While few studies included control groups, the data point to an increased capacity among LTMs for body- and self-boundary dissolution (Nave et al., 2021) and experiences of “Timelessness” and “Spacelessness” during meditation (Berkovich-Ohana et al., 2013). Body dissolution experiences were linked to five distinct dimensions: passive agency, non-locality, non-dual perception, formless attention, and body imperceptibility (Nave et al., 2021). Still, further research is needed to delineate trait effects from trait–state interactions. Lastly, LTMs’ evidence increased rational decision-making (Kirk et al., 2011) and decision thresholds, particularly for incongruent cues (van Vugt & van den Hurk, 2017), suggesting heightened objectivity and deliberateness before acting.

To explore whether meditation experience yielded incremental cognitive benefits, associations between meditative expertise and biobehavioral cognitive outcomes were observed in pain perception (Grant & Rainville, 2009), anxiety (Chen et al., 2018), NSCs, specifically phenomenological self-boundary dissolution, non-dual awareness, attentional broadening, and volitional agentic control (Nave et al., 2021), and introspective accuracy (Fox et al., 2012). Others found no such associations, as in the cases of pain perception (Grant et al., 2010), pain catastrophizing (Zorn et al., 2020), and time production, even though the latter directly compared different expertise levels (Berkovich-Ohana et al., 2011). Structural and functional brain data provide partial support for dose-dependent effects, with increased cortical thickness in pain and emotion-related regions (ACC, S1; Grant et al., 2010), as well as decreased BOLD activation in pain-related (dACC, thalamus, insula (specifically left anterior insula; Lutz et al., 2013)) and PFC areas (dlPFC, med-PFC/OFC; Grant et al., 2011). Meditation retreat hours also predicted reduced amygdala BOLD activation to negative stimuli (Kral et al., 2018), though no significant link with meditation experience was found in research investigating BOLD activation to emotional pictures (Taylor et al., 2011) and in research of compassion meditation (Lutz et al., 2008a). In summary, while dose–response effects appear plausible, they are inconsistently assessed, limiting insight into the temporal dynamics of meditation-related cognitive development. Future research should rigorously quantify predictor variables and systematically analyze dose–response effects, including potential non-linearities between meditative dose and development, while also considering other moderating factors when encountering diverging findings (Cearns & Clark, 2023; Cooper et al., 2022; Galante et al., 2023; Lindström et al., 2023).

Among meditation-activity specific effects, the most robust findings emerged in pain perception, favoring *observing-and-releasing* practices. This technique may enhance cognitive decentering, a core mechanism of meditation-induced pain relief (Zorn et al., 2021), which has also been linked to psychological flexibility and the quality of functioning in chronic pain populations (McCracken et al., 2013). Decentering has been proposed as critical for both non-conceptual cognition (e.g., non-dual awareness) and cognitive–affective reappraisal (Garland & Fredrickson, 2019; Thompson, 2023). Qualitative data further support this trajectory by revealing an increase in decentering and reappraisal as meditation experience accumulates (Poletti et al., 2021). For example, novice meditators tend to engage in experiential avoidance and perceive pain as purely physical, while LTMs develop patterns of reappraisal, openness, and curiosity, ultimately viewing pain as a mental construct that can be harnessed for altruistic purposes. These and other findings suggest that meditation may facilitate perceptual recontextualization through enhanced sensory–affective decoupling and cognitive–sensory integration, leading to improved sensory clarity and equanimity (Garland et al., 2015; Zeidan et al., 2012; Zorn et al., 2021). Similarly, preliminary evidence points to *observing-and-releasing* practices as particularly efficacious for promoting self- and boundary-dissolution, likely due to their cultivation of attentional disengagement and agentic passivity (Nave et al., 2021). This aligns with predictive processing accounts of meditation research, which propose that attentional relaxation and “in-action” reduces top-down precision weighting and consequently self-evidencing, ultimately flattening the predictive hierarchy and giving rise to non-dual awareness and minimal phenomenal experiences, characterized by a unified experience of “here” and “now,” in which the dichotomy of perceiver and perceived has collapsed (Laukkonen & Slagter, 2021; Lutz et al., 2019; Mago et al., 2024).

In the final discussion section, we will synthesize our findings through a neurophenomenological lens. Drawing on self-processing frameworks, we explore how the cognitive domains reviewed interrelate dynamically across their behavioral, phenomenal, and neural correlates, and how LTMs may exemplify an embodied, dynamical gestalt of mindfulness.

### 4.3 Self-processing and the brain in long-term meditators

Extensive research has synthesized meditation-related brain changes (Cooper et al., 2022; Fox et al., 2016; Hölzel et al., 2011; Prakash et al., 2024; Rahrig et al., 2022; Sezer et al., 2022; Tang et al., 2015; Treves et al., 2024a; Vago & Silbersweig, 2012), identifying key neural correlates. Despite evidencing converging findings, a gap remains in consolidating neural, behavioral, and phenomenological results (Hölzel et al., 2011). This is not only due to methodological variability but also due to the deeply interrelated processes under study (Hölzel et al., 2011; Vago & Silbersweig, 2012). A behavior may be associated with several neural processes, which, in turn, dynamically change based on population, meditative activity, and experience.

To address this complexity, prior work relied on a self-processing lens, in which the self-model serves as a non-reductive interdisciplinary ground for integration (Cooper et al., 2022; Vago & Silbersweig, 2012). Here, we draw on the Pattern Theory of Self (Gallagher, 2013, 2021, 2024; Gallagher & Daly, 2018; Gallagher et al., 2023)—as one such model—not as a definitive explanatory framework, but as a heuristic for the synaptic integration to help organize the diverse findings reviewed in this paper.

**Fig. 2.**
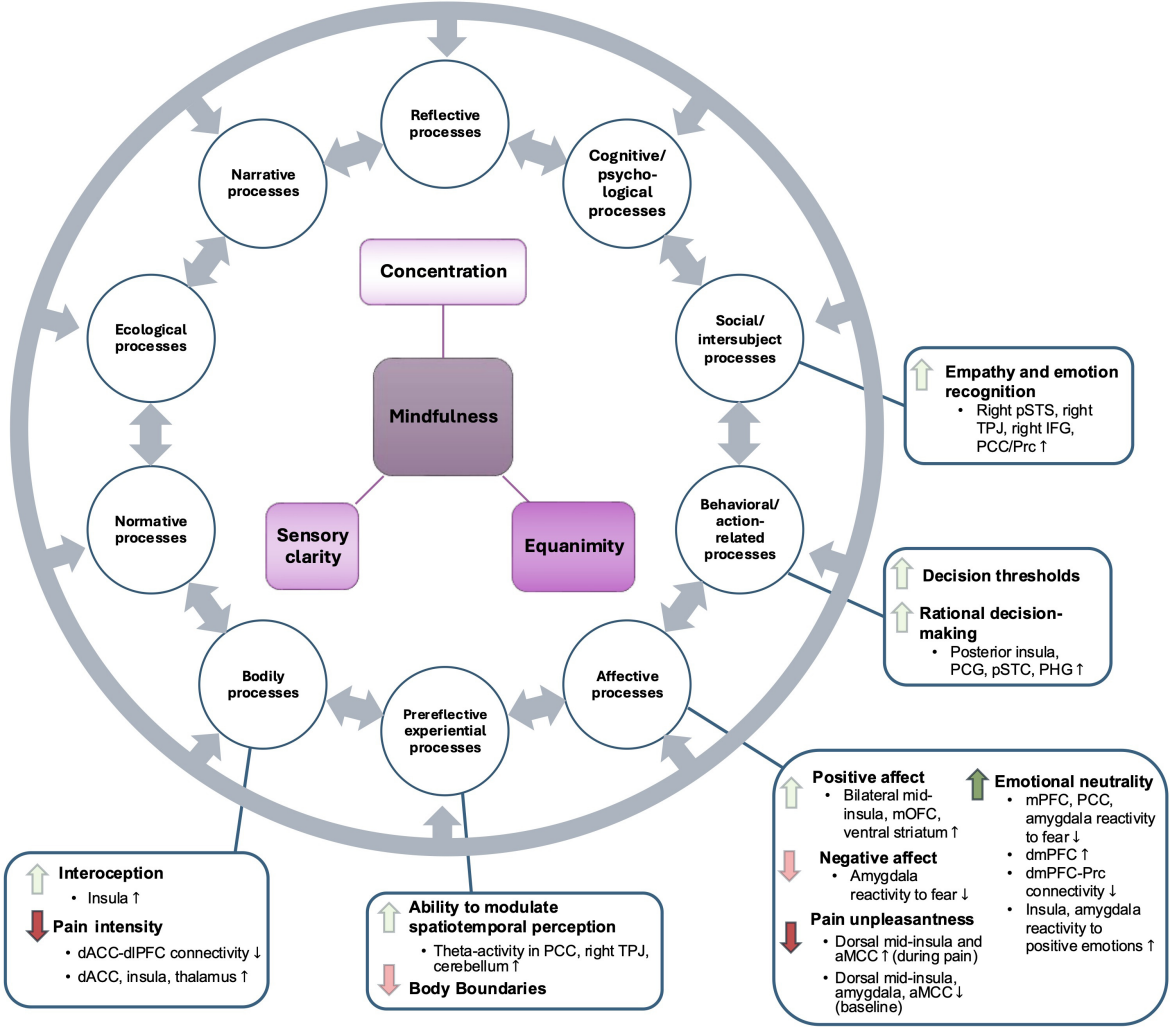
The cognitive neurobehavioral results of LTMs contextualized in the pattern theory of self. *Note*. The 10 circles graphically depict the pattern theory of self, where each self-process dynamically relates to all other processes, creating an overarching dynamical gestalt (Gallagher, 2021). The central part of the figure provides a non-comprehensive description of the dynamical gestalt of LTMs. We propose a skill-based model of mindfulness as a suitable way to describe changes in the gestalt and, consequently, the self-pattern, with less transparency in the three factors of mindfulness indicating enhanced robustness of evidence (Young, 2016). The dynamical gestalt does not causally influence the pattern; rather, the pattern itself embodies the gestalt through its processes and relations (e.g., rational decision-making as a behavioral expression of equanimity). The dynamical gestalt and the evidence for different self-processes are limited by the scope of the review and do not represent an absolute depiction of LTMs. The boxes summarize the behavioral and neural results for individual constituents in the self-pattern. The arrows indicate the directionality of change, with gray arrows denoting preliminary support and dark arrows representing stronger evidence. Small arrows show the direction of connectivity or activation changes for neural findings. aMCC: anterior midcingulate cortex; dACC: dorsal anterior cingulate cortex; dmPFC: dorsomedial prefrontal cortex; dlPFC: dorsolateral prefrontal cortex; IFG: inferior frontal gyrus; mPFC: medial prefrontal cortex; mOFC: medial orbitofrontal cortex; PCC: posterior cingulate cortex; PCG: postcentral gyrus; PHG: parahippocampal gyrus; Prc: precuneus; pSTC: posterior superior temporal cortex; pSTS: posterior superior temporal sulcus; TPJ: temporoparietal junction.

The theory of self is a non-reductionistic, interdisciplinary enactivist account positing that the self is a dynamic pattern constituted by a set of factors or processes, which do not have any strictly necessary conditions but together form a sufficient configuration (Gallagher, 2013, 2021). The self-pattern characterizes a dynamical gestalt, such that the weighting of each constituent depends on and influences the relations among the others. The theory is compatible with Buddhist accounts of the self (Gallagher & Daly, 2018; Gallagher et al., 2023) and has been applied to psychiatry to conceptualize psychopathology as a self-pattern disruption (Gallagher, 2024). Refer to Table 2 for a detailed description of these processes, and Figure 2 for a summary of our findings in relation to the self-pattern.

**Table 2.** Dynamical processes of the self-pattern.

Elements of the pattern	Brief description
Bodily processes	Including bio-systemic processes related to motoric, autonomic, endocrine, enteric, immune, interoceptive functions, supporting homeostasis and a basic distinction between self and non-self.
Prereflective experiential processes	Pre-reflective self-awareness is a structural feature of consciousness constrained by bodily factors; it includes a sense of ownership or mineness, and a sense of agency for intentional action. These processes form what is sometimes called the minimal self (Gallagher, 2000; Gallagher & Zahavi, 2020, 2021).
Affective processes	Including factors ranging from basic bodily affects (e.g., hunger, fatigue) to typical emotion patterns, existential feelings, and moods (Newen et al., 2015; Ratcliffe, 2008).
Behavioral/action-related processes	Our actions and habitual behaviors which contribute significantly to our self-identity and character (Dewey, 1922; Verplanken & Sui, 2019).
Social/intersubjective processes	Ranging from a basic capacity for attuning to others (de Waal, 2004; Reddy, 2008; Rochat, 2011; Trevarthen, 1979) to a more developed consciousness of self as distinct from others (Mead, 1913; Sartre, 1969; Taylor, 1989).
Cognitive and psychological Processes	Standard theories of personal identity highlight psychological continuity and memory (e.g., Shoemaker, 2011); self-related cognitive processes include concepts, beliefs, cognitive dispositions, and personality traits.
Reflective processes	“The ability to consciously reflect on one’s experiences and actions, closely related to notions of autonomy and moral personhood, including the capacity to evaluate and form second-order volitions about one’s desires” (Gallagher, 2021, p. 129; see Frankfurt, 1988; Taylor, 1989).
Narrative processes	Narrative self-interpretation recursively reflects other processes in the self-pattern. Theories of self-narrative may include strong claims about how narratives constitute the self (Dennett, 1991; Ricoeur, 1992; Schechtman, 2011).
Ecological processes	“Our embodied-situated actions engage with (and sometimes incorporate) artifacts, instruments, bits and structures of the environment in ways that define us and scaffold our identities. Situations shape who we are, and affordances define our possibilities” (Gallagher, 2021, p. 129).
Normative processes	Including social and cultural features that are expressed in value-determining norms that define oughts, obligations, and expectations. Self-identity or the sense of who one is is shaped by everything that comes along with one’s profession, one’s religion, social status, the various roles involved in marriage, in parenting, in friendship, as well as constraints imposed by gender, race, and economic circumstances, for better or worse.

*Note.* Adapted from Gallagher (2021, p. 128).

Two overarching results stand out. First, there is no simple mapping between neural and behavioral findings: similar brain regions are implicated in multiple, distinct behavioral and self-related processes, cautioning against reverse inference, especially in different populations like this one (Jack et al., 2019). Second, despite this, our findings arguably converge toward an embodied, dynamical configuration of mindfulness. From this perspective, different levels of explanation—behavioral, neural, and phenomenal—become complementary. Accordingly, when practicing meditation, initiating changes to the self-gestalt influences the individual constituents, while modifying these processes can adjust the overall self-pattern by reassigning weights and values to these components (Gallagher, 2021). To understand this dynamical gestalt of an LTM, it may be helpful to consider systematic classifications of mindfulness, as extended practice may shape the pattern according to these principles. Figure 2 provides a non-comprehensive characterization of the dynamical configuration of LTMs based on our reviewed evidence from Young’s mindfulness model (Young, 2016). Crucially, the sub-facets of mindfulness—concentration, sensory clarity, and equanimity—are embedded and embodied within the self-pattern, rather than emerging from it as the figure might imply. For example, rational decision-making is one expression of equanimity, not a separate faculty. Consequently, the dynamical gestalt is the pattern and its relations.

Supporting prior work linking mindfulness to enhanced body awareness (Treves et al., 2019), our results indicate increased sensory clarity in LTMs. This clarity spans bodily, pre-reflective experiential, affective, behavioral/action-related, and social intersubjective processes, and is associated with increased activation in core SN regions, such as the insula and dACC, as well as the right TPJ, thalamus, and aMCC (see Fig. 2; Grant et al., 2011; Kirk et al., 2011; Lutz et al., 2013). Consistent findings of insula activation in meditation research (Cooper et al., 2022; Fox et al., 2016; Hölzel et al., 2011; Sezer et al., 2022; Tang et al., 2015; Vago & Silbersweig, 2012; Young et al., 2018) support its central role in body awareness (Treves et al., 2024a). Yet nuances remain: increased posterior but decreased anterior insula activation may reflect heightened interoceptive but decreased affective processing (Kirk et al., 2011), and rightward lateralization suggests a shift toward perceptual rather than cognitive empathy (Fan et al., 2011; Lutz et al., 2008a).

Thus, the insula may serve as a hub for sensory clarity and for gaining differential insight into the various layers of self-processing (Bodhi, 2011; Sharf, 2014). However, further research is needed to clearly differentiate meditation’s effects on interoceptive accuracy versus perceptual sensory acuity, and their relationship with insula function. Studies on perceptual processing in LTMs revealed reduced habituation and enhanced sensitivity, implying reduced top-down and increased bottom-up processing (Antonova et al., 2015; Berkovich-Ohana et al., 2013; Fucci et al., 2018; Kasamatsu & Hirai, 1966). Sensory clarity may thus enhance interoceptive awareness by improving detection, discrimination, and penetration of sensory signals (Young, 2016). This enables higher-level processes to more precisely track lower-level signals, paralleling the proposed role of the frontal-parietal control network, which shows a strong overlap in key brain regions (dACC, aMCC, TPJ; Vago & Silbersweig, 2012). For example, enhanced sensory clarity may help distinguish cognitive–evaluative from sensory–discriminant experiences, enabling the decomposition of congealed sensory–affective phenomena into their components. This is reflected in LTMs’ increased activation within the ACC, thalamus, insula, and somatosensory cortices and decreased activation in the PFC, hypothalamus, and amygdala (Grant et al., 2011; Lutz et al., 2013), in which aforementioned brain areas (e.g., ACC, insula, thalamus) are necessary but not sufficient for this deconstruction. As such, processes such as rational decision-making, emotion recognition, pain unpleasantness, and emotional neutrality may hinge on the precise parsing of sensory input, but are also shaped by broader self-related dynamics.

Stronger evidence was found for increases in equanimity, as demonstrated by changes in bodily, pre-reflective experiential, affective, behavioral/action-related, and social intersubjective processes. Specifically, LTMs displayed reduced pain sensitivity and unpleasantness, enhanced emotional neutrality, rational decision-making, and empathy. Equanimity may be defined as a state of internal balance, in which there is no suppression, nor grasping toward internal or external sensory experiences (Young, 2016). Despite disagreement on whether equanimity is enclosed within or separate from mindfulness, previous research proposed equanimity as a core outcome of mindfulness meditation (Eberth et al., 2019) as it is critically related to emotion regulation and thus well-being (Desbordes et al., 2014).

Indeed, besides changes in sensory processing, improved emotion regulation emerged as the most dominant result in our review. Previous research proposed corticolimbic decoupling at the root of emotion regulation in meditation, consequently extending to favorable changes in other areas, such as pain perception (Sezer et al., 2022). Besides changes in insula activation, we observed changes in activation in affective and pain-related areas, including the mOFC, aMCC, amygdala, and VS. PFC activation remains ambiguous, demonstrating increases primarily in the initial stages of emotion regulation (Kral et al., 2018) but not long term (Young et al., 2018). These results, however, may be too simplistic, as reduced within-DMN activation (Taylor et al., 2011), as well as increased dmPFC and decreased within-DMN connectivity (Lutz et al., 2016), suggests alternative mechanisms driving improvements in emotion regulation. The latter finding indicates areas (dmPFC) involved in attentional self-control during emotional states (Farb et al., 2007; Jin et al., 2024; Walter et al., 2009). Attention regulation, a core cognitive mechanism underlying the benefits of mindfulness meditation (Malinowski, 2013), has been associated with increased rsFC between dlPFC and PCC when not distinguishing experience levels (Sezer et al., 2022) and enhanced prefrontal and parietal surface area (Treves et al., 2024a). However, attention’s role in emotion regulation is complex and depends on the meditative practice (Lee et al., 2012).

In fact, different practices facilitate emotion regulation through different yet partially overlapping neural substrates (Fox et al., 2016; Vago & Silbersweig, 2012). Attention-based styles engage attentional- and emotion-processing areas, LKM involves the activation of emotion, affiliation, reward, and self-referential processing areas (Engen & Singer, 2015; Lee et al., 2012), and Zen practice alters mental self-processing (Taylor et al., 2011). Changes in mental self-processing influence normative self-awareness, specifically the experience of the narrative self (Northoff et al., 2006). AMs’ heightened aptitude to dissolve the narrative self and experience states of “timelessness” and “spacelessness” may reflect improved control over crucial DMN regions, such as the PCC (Berkovich-Ohana et al., 2013). Indeed, reduced PCC activation is one of the most consistent findings in meditators (Cooper et al., 2022), while mPFC effects appear more variable and may depend on meditative expertise and/or location of frontal midline activation (ventral/dorsal; Lutz et al., 2016).

These findings highlight the mutual influence of cognitive and self-related processes. For example, changes in narrative self-referential processing can improve emotion regulation and vice versa (Gallagher, 2024). This insight may offer leverage if harnessed within precision medicine, as targeting a specific self-process or its associated neural correlate may provide the potential to explore changes in the dynamic pattern configuration (Gallagher, 2024). For example, neurofeedback studies targeting DMN hubs showed improvements in broader self-pattern symptoms (Bauer et al., 2020; Zhang et al., 2023). Similarly, effects may extend to the enhancement of meditative development through non-invasive brain stimulation, where targeting self-related networks (PCC) has been found to increase equanimity and mindfulness, reduce self-interference, and potentially promote adaptive regulatory dynamics (Abellaneda-Pérez, et al., 2024; Lord et al., 2024a, 2024b).

Our results parallel the “Topographical Reorganization of Meditation” model, which maps meditational development onto three spatially nested self-processing layers: interoceptive, exteroceptive, and mental (Cooper et al., 2022). Interoceptive processing involves physiological interaction with the environment, exteroceptive processing includes proprioceptive and affective perception, and mental self-processing includes cognitive aspects of self-representation (Qin et al., 2020). Lower-level intero-exteroceptive processing is naturally non-dual, while higher-level mental processing introduces self-object dichotomies. Through meditation, non-dual intero-exteroceptive processing shifts to the foreground, revealing brain–body–environment interconnectedness, and relegates self-referential mental processing to the background, making non-dual perception explicit (Thompson & Varela, 2001; Northoff & Zilio, 2022). Our results similarly suggest enhanced lower-level intero-exteroceptive self-processing with corresponding reductions in narrative activity.

This also aligns with a review by Vago and Silbersweig (2012), as our findings indicate increased activation in networks associated with the non-conscious experiential enactive self (limbic regions, thalamus, and posterior insula) and the conscious experiential phenomenological self (anterior insula, S1, pSTS, and Prc). Reduced activation was noted in narrative self-reflective brain areas (mPFC and PCC). In Cooper’s model, low meditation proficiency corresponds to high DMN and low FPN activation. With ongoing practice, this pattern reverses and eventually stabilizes into co-active FPN and DMN. The authors hypothesized a mediating role of the SN between DMN and FPN, supported by other studies showing an inverted “U” relationship between SN activation and meditation proficiency (Brefczynski-Lewis et al., 2007; Cooper et al., 2022; Rahrig et al., 2022). While we found some evidence for increased SN activation (see also fronto-parietal control network; Vago & Silbersweig, 2012), we could not confirm or rule out this temporally sensitive reorganization. Due to space constraints, we did not review the broader attentional literature on LTMs; thus, confirming these topographical dynamics and assessing concentration in LTMs (see Fig. 2) fall beyond the scope of this review.

Taken together, these findings support the idea of a meditative neurophenomenological gestalt, wherein behavioral outcomes reflect large-scale system reorganizations rather than isolated regional changes. Different mindfulness practices can flexibly modulate core self-processes, such as attention, emotions, or self-consciousness, supporting the transition between different self-networks (Gallagher et al., 2023; Vago & Silbersweig, 2012). This suggests a dispositional reconfiguration through mindfulness, particularly in the domains of equanimity and sensory clarity.

In alignment with meditative predictive processing accounts, meditation may enhance access to more embodied cognitive–affective aspects of the self (Christoff et al., 2011; Laukkonen & Slagter, 2021; Lutz et al., 2019). Increases in well-being may then emerge from the suspension of habitual avoidant mental actions, allowing previously repressed content to surface and be acknowledged, thereby enhancing self-understanding and disrupting habitual processes that maintain negative affect (Deane et al., 2024). As a result, meditative practices may foster cognitive flexibility and epistemic access to one’s mental processes, supporting insight into causal dynamics across affective and cognitive domains. This aligns with Buddhist psychological accounts of insight into “No-Self,” where the self is seen not as a fixed entity but as a dynamic and interdependent process lacking inherent substance (Dambrun & Ricard, 2011; Lutz et al., 2019; Vago & Silbersweig, 2012).

In this context, inflexibility within self-processing, especially when cemented through rigid top-down patterns, might represent a fundamental transdiagnostic element in psychopathology that meditative practices can alleviate (Berkovich-Ohana et al., 2024; Gallagher, 2024; Gallagher et al., 2023; Giommi et al., 2023). Converging with our approach and findings, an interdisciplinary framework on meditative development “The Pattern Theory of Selflessness” suggests that meditation fosters increased self-pattern flexibility—the capacity to shift between, integrate, or temporarily suspend different elements of the self-pattern (Berkovich-Ohana et al., 2024). Reduced flexibility, by contrast, supports perseverative states such as rumination, anxiety, craving, or self-clinging. The PTSL model outlines six interrelated transformations—including consolidation, mindful awareness, decentering, and deconstruction—that iteratively reorganize the self-pattern. This dynamic process may gradually reduce reification and increase adaptive fluidity, allowing for trait-level selflessness and greater psychological resilience.

Complementing these conceptually advanced, novel neuroimaging approaches has begun to identify functional connectome signatures of meditative expertise. In particular, LTMs exhibit reduced functional gradient hierarchies and greater integration across sensorimotor, affective, and attentional networks—patterns associated with cognitive–affective decentering and indicative of a shift toward more distributed, embodied modes of cognition (Czajko et al., 2023). Gradients are characteristic spatial topographies of brain organization, indexing structural–functional–cognitive relationships, and linked with cognitive–behavioral developmental changes (Bernhardt et al., 2022; Dong et al., 2021; Huntenburg et al., 2018). This functional gradient compression between sensory and transmodal brain regions is also observed in acute psychedelic-induced states (Erritzoe et al., 2024). These states exhibit significant overlap with meditative neurophenomenology, particularly regarding changes in self-consciousness (Millière et al., 2018).

Lastly, consistent with prior models, cognitive improvements from meditative expertise may not stem from enhanced cognitive control, but rather from shifts in the brain’s default activation patterns (Cooper et al., 2022; Tang et al., 2015). Persistent perceptual shifts toward non-dual awareness, along with concomitant changes in default cognitive processing, such as reduced repression and enhanced equanimity, sensory clarity, and perhaps concentration, may be the most distinguishing factors between advanced and beginner meditators. While short-term practice can induce meaningful cognitive and clinically effects (Ainsworth et al., 2013; Tang et al., 2007; Zeidan et al., 2010a; 2010b), sustained skillful training may be necessary to gain access to fundamental changes in perceptual (Cooper et al., 2022; Josipovic, 2010; Josipovic, 2021), social (Singleton et al., 2021; Trautwein et al., 2016; Williams et al., 2016), and emotional processing (Sahdra et al., 2011) as described by ancient soteriological traditions.

To advance the field, we propose future research focus on (1) employing a unified framework to assess predictors of meditative development and mastery (Galante et al., 2023), (2) systematically gathering phenomenological data and its associated practices (Sparby & Sacchet, 2022), (3) translating and testing experience patterns from different stages of meditative development into neuroscientific hypotheses (Wright et al., 2023), and (4) investigating topological and topographical changes associated with cognitive functions across timescales to illuminate the interrelated, dynamic, and nested nature of cognitive and meditative development.

## 5 Summary and Conclusion

This review synthesized the biobehavioral cognitive outcomes of LTMs, indicating preliminary evidence of changes in perception, emotion processing, self-awareness, and higher-order functions. Specifically, LTMs exhibited increased interoceptive awareness, rational decision-making, emotional neutrality, and self-body boundary dissolution, alongside reduced pain perception and enhanced pre-reflective self-awareness. Correlations between practice experience and cognitive outcomes suggest a dose–response relationship, though limitations preclude firm conclusions.

To address these issues, unitive meditative frameworks are needed, incorporating systematic assessment of predictor variables, sociocultural context, and clear delineation of acute (state) and habitual (trait) factors, to improve consistency and interpretability (Galante et al., 2023). Bridging phenomenological reports with brain data will require interdisciplinary collaboration and integrative models such as the Thin Model (Wright et al., 2023). Such efforts will aid the understanding of meditative adverse events (Lindahl et al., 2017, 2020) and improve evidence-based applications for well-being or psychopathology.

LTMs appear to exhibit a distinct neurophenomenological profile of mindfulness, characterized by altered processing in affective, attentional, sensorimotor, and self-referential brain areas, alongside enhanced cognitive integration. Phenomenological transitions from implicit to explicit non-dual awareness may signal a shift in embodied cognition in which top-down mental self-processing recedes and bottom-up experiential self-processing is foregrounded. More specifically, meditative expertise was embedded within bodily, pre-reflective experiential, affective, behavioral/action-related, and intersubjective self-processes and their dynamic interactions, potentially reflecting enhanced equanimity and sensory clarity.

Future research should test and refine neurophenomenological models, such as the “Topographical Reorganization of Meditation,” by mapping topographical and temporal changes across cognitive domains and meditative populations. Ultimately, the field must adopt a more comprehensive framework of advanced meditation that integrates both development and phenomenology, moving toward a science of embodied mastery rather than relying solely on “long-term practice” as measured by hours completed (Sacchet et al., 2024). Despite these concerns, preliminary results provide converging functional, phenomenological, and neural associations, underscoring the promise of contemplative neuroscience.

## Data Availability

This review did not involve the collection or analysis of original data. No data or code are available.

## Glossary

**ACC** anterior cingulate cortex

**aMCC** anterior mid-cingulate

**ACC** anterior cingulate cortex

**dACC** dorsal anterior cingulate cortex

**DDM** drift diffusion model

**DMN** default mode network; associated with self-referential thought, autobiographical memory, and mental time travel

**dmPFC** dorsomedial prefrontal cortex

**dlPFC** dorsolateral prefrontal cortex

**FPN** frontoparietal network

**IFG** inferior frontal gyrus

**IPS** intraparietal sulcus

**LKM** loving-kindness meditation

**OFC** orbitofrontal cortex

**PCC** posterior cingulate cortex

**PCG** postcentral gyrus

**PFC** prefrontal cortex

**Prc** precuneus

**PHG** parahippocampal gyrus

**pSTC** posterior superior temporal cortex

**pSTS** posterior superior temporal sulcus

**MCC** mid-cingulate cortex

**MFG** middle frontal gyrus

**mOFC** medial orbitofrontal cortex

**mPFC** medial prefrontal cortex

**mindfulness meditation** mindfulness meditation comprises *samatha* and *vipassanā*, both *observing* type practices, with the former based on *focusing* attention and the latter a combination of *focusing* and *releasing* activities

**nondual awareness** a state of consciousness in which the distinction between observer and observed is reduced, resulting in what is thought of as more direct awareness of awareness itself. This non-conceptual state is thought to be cultivated through advanced meditation and may reflect reflexive knowing without subject–object duality.

**Nyingma Tradition** a specific tradition rooted in Tibetan Buddhism that is known for its cultivation of compassion, loving-kindness, and altruism.

**rACC** rostral anterior cingulate cortex

**rsFC** resting-state functional connectivity

**S1** primary somatosensory cortex

**S2** secondary somatosensory cortex

**Sant Mat mediation** Sant Mat meditation is centered on loving-kindness meditation and encompasses practicing unwavering compassion as a core focus.

**SFG** superior frontal gyrus

**SN** salience network

**Tai Chi Chuan** Tai chi chuan is a form of self-defense training that emphasizes purposeful movement, making it consistent with the concept of an active form of meditative practice

**Therāvada Buddhism** Theravāda practices include investigative *observe-and-release* and *focus-and-observe* activities as well as compassion/LKM (*mettā*) practices

**Tibetan Buddhism** Tibetan Buddhism practices integrate *receptive observation-based* practices with *active* visualizations, mantra recitations, and compassion-based meditations such as Tonglen, aimed at transforming the self and developing universal compassion.

**TPJ** temporoparietal junction

**Transcendental meditation** Transcendental meditation is an active meditation technique using the silent repetition of a mantra while remaining in a focused state

**vmPFC** ventromedial prefrontal cortex

**VS** ventral striatum

***Vipassanā*** a mediation type utilizing *focused observing*, and *observe-and-release* practices.
